# Lysyl hydroxylase 1 (LH1) deficiency promotes angiotensin II (Ang II)-induced dissecting abdominal aortic aneurysm

**DOI:** 10.7150/thno.65277

**Published:** 2021-09-21

**Authors:** Hao Li, Haochen Xu, Hongyan Wen, Hongyue Wang, Ranxu Zhao, Yingying Sun, Congxia Bai, Jiedan Ping, Li Song, Mingyao Luo, Jingzhou Chen

**Affiliations:** 1 State Key Laboratory of Cardiovascular Disease, Fuwai Hospital, National Center for Cardiovascular Diseases, Chinese Academy of Medical Sciences and Peking Union Medical College, Beijing 100037, China.; 2 Department of Pathology, Fuwai Hospital, National Center for Cardiovascular Diseases, Chinese Academy of Medical Sciences and Peking Union Medical College, Beijing 100037, China.; 3 State Key Laboratory of Cardiovascular Disease, Center of Vascular Surgery, Fuwai Hospital, National Center for Cardiovascular Disease, Chinese Academy of Medical Sciences and Peking Union Medical College, Beijing 100037, China.; 4 Department of Vascular Surgery, Fuwai Yunnan Cardiovascular Hospital, Affiliated Cardiovascular Hospital of Kunming Medical University, Kunming, 650102, China.; 5 National Health Commission Key Laboratory of Cardiovascular Regenerative Medicine, Fuwai Central-China Hospital, Central-China Branch of National Center for Cardiovascular Diseases, Zhengzhou 450046, China.

**Keywords:** angiotensin II, dissecting abdominal aortic aneurysm, lysyl hydroxylase, mice, thrombospondin-1

## Abstract

**Rationale:** The progressive disruption of extracellular matrix (ECM) proteins, particularly early elastin fragmentation followed by abnormalities in collagen fibril organization, are key pathological processes that contribute to dissecting abdominal aortic aneurysm (AAA) pathogenesis. Lysyl hydroxylase 1 (LH1) is essential for type I/III collagen intermolecular crosslinking and stabilization. However, its function in dissecting AAA has not been explored. Here, we investigated whether LH1 is significantly implicated in dissecting AAA progression and therapeutic intervention.

**Methods and Results:** Sixteen-week-old male LH1-deficient and wild-type (WT) mice on the C57Bl/6NCrl background were infused with angiotensin II (Ang II, 1000 ng/kg per minute) via subcutaneously implanted osmotic pumps for 4 weeks. Ang II increased LH1 levels in the abdominal aortas of WT mice, whereas mice lacking LH1 developed dissecting AAA. To evaluate the related mechanism, we performed whole-transcriptomic analysis, which demonstrated that LH1 deficiency aggravated gene transcription alterations; in particular, the expression of thrombospondin-1 was markedly upregulated in the aortas of LH1-deficient mice. Furthermore, targeting thrombospondin-1 with TAX2 strongly inhibited the proinflammatory process, matrix metalloproteinase (MMP) activity and vascular smooth muscle cells (VSMCs) apoptosis, ultimately decreasing the incidence of dissecting AAA. Restoration of LH1 protein expression in LH1-deficient mice by intraperitoneal injection of an adeno-associated virus normalized thrombospondin-1 levels, subsequently alleviating dissecting AAA formation and preserving aortic structure and function. Consistently, in human AAA specimens, decreased LH1 expression was associated with increased thrombospondin-1 levels.

**Conclusions:** LH1 deficiency contributes to dissecting AAA pathogenesis, at least in part, by upregulating thrombospondin-1 expression, which subsequently enables proinflammatory processes, MMP activation and VSMCs apoptosis. Our study provides evidence that LH1 is a potential critical therapeutic target for AAA.

## Introduction

Abdominal aortic aneurysms (AAAs) are devastating age-related vascular conditions occurring within the aortic wall, which if left unchecked, lead to extensive dilatation of the abdominal aorta and progress to life-threatening aortic rupture 1, 2. Despite significant improvements in the diagnosis and treatment, such as surgical intervention and endovascular repair of AAA, the rate of morbidity and mortality from aortic rupture remains high 3. Notably, pharmacologic prevention is ineffective other than corrective surgery for these conditions to date 4, highlighting the need to expand our knowledge of the molecular and cellular mechanisms responsible for the vascular degeneration underlying AAA to provide new insights into the development of new therapeutic approaches.

AAA occurs as a result of drastic changes to the structure of the aortic wall. These changes occur mainly due to infiltration of inflammatory cells, loss of vascular smooth muscle cells (VSMCs) and destruction of extracellular matrix (ECM) proteins 5. Elastin and collagen are the major components of the aortic ECM and organize the abdominal aortic wall 6. Early elastin fragmentation followed by abnormalities in collagen fibril organization are hallmarks of AAA pathology and are considered crucial for aortic dilation and eventual rupture 7, 8.

Lysyl hydroxylase 1 (LH1), encoded by the procollagen-lysine, 2-oxoglutarate 5-dioxygenase 1 (*PLOD1*) gene, is an enzyme that plays an important role in hydroxylation of lysyl residues in Xaa-Lys-Gly. LH1 catalyzes the hydroxylation of lysyl residues in collagen-like peptides, which is essential for the stability of the intermolecular cross-links that provide the collagen fibrils with their tensile strength and mechanical stability 3, 9. Notably, LH has three isoforms (LH1, 2, and 3); both LH1 and LH3 could have substrate preferences, and LH1 prefers type I/III collagen 10. Deficiency of LH1 in humans due to mutations of the *PLOD1* gene has been identified as the pathogenic cause of the kyphoscoliotic subtype (subtype VIA) of Ehlers-Danlos syndrome (EDS), which is a rare heterogeneous group of heritable ECM disorders characterized by progressive kyphoscoliosis, congenital muscular hypotonia, joint hypermobility and severe skin fragility 9. Notably, it has been reported that life-threatening vascular incidents such as aortic dilation and rupture occur in EDS patients 11. Furthermore, *Plod1^-/-^* mice show an increased risk of aortic rupture compared with wild-type (WT) mice 3. However, whether LH1 deficiency plays a causal role or directly participates in the pathogenesis of AAA remains elusive.

An angiotensin II (Ang II)-infused mouse model is currently ubiquitously used for preclinical aneurysm research. However, increasing evidence indicates that Ang II-induced lesions in mice should be considered pseudoaneurysms or dissecting AAA, as relevant differences are observed in human AAA 12-14. To avoid confusion, we therefore referred to Ang II-induced lesions as 'dissecting AAAs' rather than AAAs in the present study.

This study aimed to evaluate the role of LH1 in aneurysm pathogenesis by using AAA specimens from patients and Ang II-induced dissecting AAA LH1-deficient (*Plod1^-/-^*) model mice. We report a novel protective function for LH1 against dissecting AAA; LH1 absence results in thrombospondin-1 (encoded by *Thbs1*) expression upregulation, promotion of the proinflammatory process, increased matrix metalloproteinase (MMP) activity, and severe VSMC apoptosis in the abdominal aorta, ultimately leading to dissecting AAA formation.

## Materials and methods

The data that support the findings of this study are available from the corresponding author upon reasonable request.

### Human subjects

Human AAA specimens from the anterior region of the aortic wall were procured from 6 patients undergoing open AAA surgery at Fuwai Hospital, National Center for Cardiovascular Diseases, Beijing, China. The patients were diagnosed with AAA by repeated ultrasonography or CT angiography. AAA patients with rare heritable disorders such as Marfan syndrome, Loeys-Dietz syndrome or EDS were excluded. Patients with atherosclerosis were also excluded. Non-AAA aortic tissues were resected from the aortic roots of 6 patients who underwent heart transplantation for hypertrophic cardiomyopathy or dilated cardiomyopathy and did not have an AAA. After being harvested, the aortic tissues were immediately placed in formalin, embedded in paraffin, and cut into serial sections (7 μm thick) for further histological analysis. The study protocol was reviewed and approved by the Human Ethics Committee of Fuwai Hospital (2014CB541601), and the study was conducted in accordance with the principles of Good Clinical Practice and the Declaration of Helsinki. All participants gave written informed consent. Clinical information regarding the human patients is provided in Table S1 in the Supplementary Material.

### Animals' preparation

The animal experiments were approved by the Committee of Fuwai Hospital on the Ethics of Animal Experiments and complied with the National Institutes of Health's Guide for the Care and Use of Laboratory Animals, and the manuscript adheres to the Animal Research: Reporting of *In vivo* Experiments (ARRIVE) guidelines for reporting animal experiments. Male WT C57Bl/6NCrl mice were purchased from Beijing Vital River Laboratory Animal Technology Co., Ltd. (Beijing, China). *Plod1^-/-^* mice were generated on the C57Bl/6NCrl background by Biocytogen Co., Ltd (Beijing, China) as previously described 3. WT and *Plod1^-/-^* mice were first bred to generate *Plod1^+/-^* mice, and male and female *Plod1^+/-^* mice were further interbred to generate WT and *Plod1^-/-^* littermates, which were used for the studies. The primer sequences used for genotyping were as follows: F-5'-CTGAGTGCTAGGATTTGGGCAGACC-3', R-5'-ATAAAGTCTAGCCCCAGCTCCCAAAAG-3' and R-5'-AGGGAACCAGTCCTTTCCAGTCTGA-3'.

Only male mice were studied because of the low incidence of Ang II-induced dissecting AAA in female mice, as detailed in a statement by the Arteriosclerosis, Thrombosis, and Vascular Biology Council 15. All mice were housed in a specific-pathogen-free environment on a 12 h/12 h light-dark cycle and fed a rodent diet* ad libitum*. The animals were randomly assigned to experimental groups by using the random number generator function of SPSS. Investigators blinded to the treatment groups evaluated the outcomes of all mice and performed the analysis.

### Peptide synthesis

The TAX2 peptide (CEVSQLLKGDAC) and the scrambled control peptide (LSVDESKAQGIL) were synthesized by SciLight Biotechnology Co. (Beijing, China) with a purity of 98% or higher (acetate salt was used as a counter-ion) and then analyzed by high-performance liquid chromatography (HPLC) followed by matrix-assisted laser desorption/ionization time-of-flight mass spectrometry (MALDI-TOF MS) analysis, as previously described 16. TAX2 has been shown to bind to thrombospondin-1 with comparable affinity and selectively prevents the thrombospondin-1:CD47 interaction.

### Construction of adeno-associated virus (AAV) serotype 9 vectors for *Plod1* overexpression

To induce exogenous expression of LH1 *in vivo*, we constructed a pAAV9-LH1-p2A-GFP vector (AAV-LH1) by inserting mouse LH1 cDNA into pAAV9-p2A-GFP between two ITRs (ViGene Biosciences, Shandong, China). An AAV expressing GFP (AAV-GFP) was simultaneously prepared as a control vector.

### Mouse model of dissecting AAA

The methods used to induce dissecting AAA have been described elsewhere 17. Briefly, mice were treated with Ang II (1000 ng/kg per min; Sigma-Aldrich, MO, USA) or saline for 4 weeks via osmotic pumps (Model 2004; Durect Corporation, CA, USA) that were implanted subcutaneously in 16-week-old male WT and *Plod1^-/-^* mice under anesthesia with 3% isoflurane in an O_2_ mixture (1 L/min). The mice were observed daily for normal behavior or sudden death. The mice that died were immediately necropsied to determine the cause of death.

To investigate whether thrombospondin-1 is the key mediator of dissecting AAA progression in the absence of LH1, *Plod1^-/-^* mice were intraperitoneally injected with 100 μL of TAX2 peptide (10 mg/kg) or scrambled control peptide (10 mg/kg) 3 times a week during the 4-week Ang II infusion period.

To further corroborate that LH1 is a potential critical therapeutic target for dissecting AAA, *Plod1^-/-^* mice were intraperitoneally injected once with 2×10^11^ AAV-LH1 or AAV-GFP particles in a total volume of 200 µl of supernatant. Three weeks later, the mice underwent Ang II infusion for 4 weeks.

### Ultrasound imaging

A high-resolution Vevo 2100 system (VisualSonics Inc., Toronto, Canada) was used for ultrasound imaging of the aorta. In brief, the mice were first anaesthetized with 3.0% isoflurane mixed with oxygen (100%, airflow velocity: 1 L/min); then, after the pain reflex disappeared, the heart rate stabilized to 400 to 500 beats per min. A high-frequency array probe with a center frequency of 40 MHz (Vevo MS550D) was used to collect B-mode images of the abdominal aorta. *In vivo* aortic stiffness was measured locally in the abdominal aorta by the pulse-wave velocity (PWV) technique using Vevo Vasc software as described previously 18.

Distensibility of an artery segment is a reflection of the mechanical stress affecting the arterial wall during the cardiac cycle. Stress was defined as the difference in systolic blood pressure (SBP) and diastolic blood pressure (DBP), and strain was defined as the artery system's response. Distensibility was calculated using the following algorithms, as previously described 19:

- Strain as the amount of deformation relative to the unstressed state and expressed as percent change in the arterial diameter: strain = (SD - DD)/DD, where SD is the systolic aortic diameter and DD is the diastolic aortic diameter;
- Stiffness (β) as stress (SBP - DBP)-to-strain ratio: β = ln(SBP/DBP)/strain;
- Distensibility was 1/β and adjusted to intima-media thickness (IMT): Distensibility = 1/[ln(SBP/DBP)/strain × IMT].

### Blood pressure measurement

SBP and DBP were measured in conscious mice at baseline and at days 7, 14 and 28 after pump implantation using tail-cuff plethysmography (BP-2010A, Softron, Japan). Briefly, the mice were acclimated for 2 days to restraint tubes and trial measurements. On the third day, 5 individual blood pressure measurements (technical replicates) were taken, and the mean was used for each animal. Blood pressure was measured by investigators blinded to the experimental groups.

### Histology and immunohistochemistry

After the mice were euthanized and perfused with 4% paraformaldehyde, the entire aorta was excised and visualized by stereomicroscopy (V8, Zeiss, Germany). Subsequently, the suprarenal abdominal aortas were dissected and cut into serial cryosections (7 μm thick, 300 μm apart). Sections of the abdominal aorta were subjected to hematoxylin and eosin (H&E), Verhoeff-Van Gieson (VVG) (DC0047, Leagene, China), Masson trichrome (DC0033, Leagene, China) and picrosirius red (PSR) (DC0041, Leagene, China) staining for histoarchitectural evaluation of the aneurysms. For immunohistochemical staining, sections obtained from mice and humans were blocked with 1% bovine serum albumin and 5% normal donkey serum in PBS and incubated with a combination of primary antibodies overnight at 4 °C. The sections were then rinsed and incubated with an Alexa Fluor 594-conjugated secondary antibody. The nuclei were stained with DAPI. The following primary antibodies were used: α-smooth muscle-Cy3™ (α-SMA) (1:1000, clone 1A4, C6198, Sigma-Aldrich, USA), rat anti-LH1 (1:200, ab262947, Abcam, MA, USA), rat anti-thrombospondin-1 (1:200, ab1823, Abcam, MA, USA) and anti-F4/80 (1:200, ab6640, Abcam, MA, USA). The following secondary antibodies were used: Alexa Fluor 594-conjugated donkey anti-goat IgG (1:1000, ab150132, Abcam, MA, USA) and Alexa Fluor 594-conjugated donkey anti-rat IgG (1:1000, ab150156, Abcam, MA, USA). All of the images were obtained under a Leica SP8 laser-scanning confocal microscope and a Leica DM6000B microscope. Terminal deoxynucleotidyl transferase-mediated dUTP nick-end labeling (TUNEL) staining, (Sigma-Aldrich, MO, USA) was performed using an *in situ* cell death detection kit. Briefly, sections were incubated with TUNEL mixture according to the manufacturer's instructions. We assessed the number of TUNEL^+^ cells using ImageJ software.

### Electron microscopy of mouse aortic tissue samples

For transmission electron microscopy (TEM), samples were prepared at the Department of Pathology, Fuwai Hospital following a modified version of the National Center for Microscopy and Imaging Research (NCMIR) methods for 3D EM 20. Images were acquired with a JEM-1400Flash transmission electron microscope (JEOL, Japan) at 80 kV.

### Western blot analysis

Abdominal aortic samples were lysed/homogenized in lysis buffer for 15 min at 4 °C and then centrifuged at 12,000 × g. The resulting supernatants were subjected to sodium dodecyl sulfate-polyacrylamide gel electrophoresis (SDS-PAGE) and blotted onto nitrocellulose membranes. The membranes were then incubated with an antibody against LH1 (1:1000, ab262947, Abcam, MA, USA) or thrombospondin-1 (1:1000, ab1823, Abcam, MA, USA), and the protein bands were visualized with SuperSignal West Dura Extended Duration Substrate (Thermo Fisher Scientific, IL, USA). The blots were subsequently probed with GAPDH (ab8245, Abcam, MA, USA) to confirm equal loading.

### UPLC-MS/MS assay for the quantification of amino acids

Amino acids, including lysine, proline, hydroxylysine and 4-hydroxyproline, were detected by UPLC-MS/MS 21. Each abdominal aortic sample (10 mg) was transferred to an Eppendorf tube using a tissuelyser with 20 μL of Optima grade water. Proteins were then precipitated using 120 μL of cold methanol containing IS mixture. After 20 min at -20 °C, samples were centrifuged at 13,000 g for 10 min, and then 10 μL of the supernatant was vortex-mixed with 70 μL of borate buffer (pH 8.6) followed by 20 μL of AccQTag Ultra derivatizing reagent solution. With further vortex mixing, the sample was incubated at 55 °C (10 min) and then diluted 1:100 with 900 μL of Optima grade water before UPLC-MS/MS analysis.

UPLC-MS/MS consisted of an Acquity UPLC binary solvent manager, sampler manager, and column manager (Waters, Milford, MA, U.S.A.) interfaced with a Xevo TQ-S tandem quadrupole mass spectrometer (Waters, Wilmslow, U.K.). The AccQ-tagged samples were individually injected on a Waters column (HSS T3 2.1 × 150 mm, 1.8 μm) with its temperature set to 45 °C. Water and acetonitrile containing 0.1% (v/v) formic acid were used as two mobile phases A and B, respectively, with a flow rate of 0.6 mL/min. A gradient elution scheme started at 4% B, held for 0.5 min before increasing to 10% over 2 min, then to 28% over 2.5 min, and finally increasing to 95% for 1 min, before returning to 4% B (1.3 min) for re-equilibration. The weak and strong washes were 95:5 water/acetonitrile (v/v) and 100% isopropanol, respectively. Electrospray ionization was performed in positive ion mode. The following generic source conditions were used: capillary voltage, 1.5 kV; source offset, 50 V; desolvation temperature, 600 °C; source temperature, 150 °C; desolvation gas flow, 1000 L/h; cone gas flow, 150 L/h; nebulizer gas, 7.0 bar; collision gas, 0.15 mL/min. Multiple reaction monitoring (MRM) has been used for quantification of screening fragment ions.

Peak determination and peak area integration were performed with the TargetLynx application package within MassLynx software (Waters Corporation) and the ApexTrak algorithm.

### Gelatin zymography

Zymography was performed as previously described 22. Briefly, equal amounts of protein from abdominal aortic samples were loaded and separated on a 10% Tris-glycine gel with 0.1% gelatin as the substrate. Then, the gel was washed and renatured with 2.5% Triton X-100 buffer. After incubation with developing buffer at 37 °C for 24 h, the gel was stained with 0.05% Coomassie R-250 dye (Sigma-Aldrich, MO, USA) for 30 min and destained. MMP2 and MMP9 activity levels were evaluated by measuring the optical density.

### Mouse RNA sequencing and functional analysis

Total RNA was extracted from the abdominal aortic samples using the RNeasy Mini Kit with an on-column DNase step (Qiagen, Hilden, Germany) according to the manufacturer's protocol. Immediately following extraction, the total RNA concentration and A260:A280 ratio of each sample were determined on a NanoDrop 2000 spectrophotometer (Thermo Fisher Scientific, IL, USA).

The TruSeq RNA Sample Preparation Kit V2 (Illumina, San Diego, CA) was used for next-generation sequencing library construction according to the manufacturer's protocols. The cDNA products were amplified, and sequencing adapters and barcodes were ligated onto fragments from each sample to create cDNA libraries ready for sequencing. Sequencing was performed by Annoroad Gene Technology Corp. (Beijing, China) using the Illumina HiSeq X Ten platform.

Clean data without ribosomal RNA reads for each sample were aligned to the reference genome using HISAT2 (v2.0.1). Then, four procedures, 'stringtie, stringtie --merge, cuffquant, and cuffnorm with default parameters, were used to reconstruct transcripts, identify novel transcripts, quantify transcripts, and normalize expression values (fragments per kilobase of transcript per million mapped reads, FPKM). For differential expression analysis, the edgeR package in R language (v3.2.1) was used to identify differentially expressed genes (DEGs). The fold change in expression between the two groups was calculated as logFC = log2 (experimental/control group). Genes with a |logFC| > 1 and q-value < 0.05 between the two groups were defined as DEGs. DEGs were hierarchically clustered to visualize the expression patterns using the ward method for the Euclidean distance matrix. The clustered gene expression profile of each group is shown as the mean of log2 (FPKM). We used UpSet plot 23, which is a new visualization technique for quantitative analysis of interaction sets, instead of a Venn diagram to present the relationships between the different groups. By using gprofiler (v1.0.0), the DEGs identified by RNA sequencing were further analyzed based on Human Phenotype Ontology. InterProScan (v5.8-49.0) and blast2GO software were used to annotate domains, gene families, and Gene Ontology (GO) functions. KOBAS (v2.0) with default parameters was used to perform KEGG pathways analysis of the genes. A hypergeometric distribution test was carried out to identify the GO functions and KEGG pathways in which DEGs were significantly enriched (q-value < 0.05) compared with the total background expressed genes. Enriched GO items and KEGG pathways were plotted by Python (v2.7.9) using the matplotlib (v1.4.3) package.

### Quantitative real-time PCR

Relative quantification by real-time PCR was performed using SYBR Green to detect PCR products in real time with the ABI PRISM 7500 Sequence Detection System (Applied Biosystems). Melting curve analysis was performed at the end of each PCR. Oligonucleotide primers were designed according to the cDNA sequences in the GenBank database using Primer Express software (Applied Biosystems) and are listed in Table S2 in the Supplementary Material.

### ELISA

The plasma levels of interleukin (IL)-6 and tumor necrosis factor alpha (TNF-α) in mice were measured using ELISA kits (R&D Systems Incorporated, MN, USA). All values were in the linear range and were calculated based on known protein concentrations.

### Statistical analysis

Quantitative results are expressed as the means ± SD. The sample distribution was determined using the Kolmogorov-Smirnov normality test, and all data showed normal distribution. Comparisons between any two groups were performed by a two-tailed unpaired Student's t-test. Two-way ANOVA followed by Bonferroni post hoc test was used to compare multiple groups using two main factors (LH1 deficiency and Ang II treatment). One-way ANOVA followed by Bonferroni post hoc test was used to compare multiple groups with one factor (time points). The rates of aneurysm formation and rupture in each group were evaluated using Fisher's exact test. The data were analyzed using GraphPad Prism (GraphPad Software Inc., CA, USA). *P <* 0.05 was considered statistically significant.

## Results

### LH1 deficiency exacerbates Ang II-induced dissecting AAA

**Figure 1:**
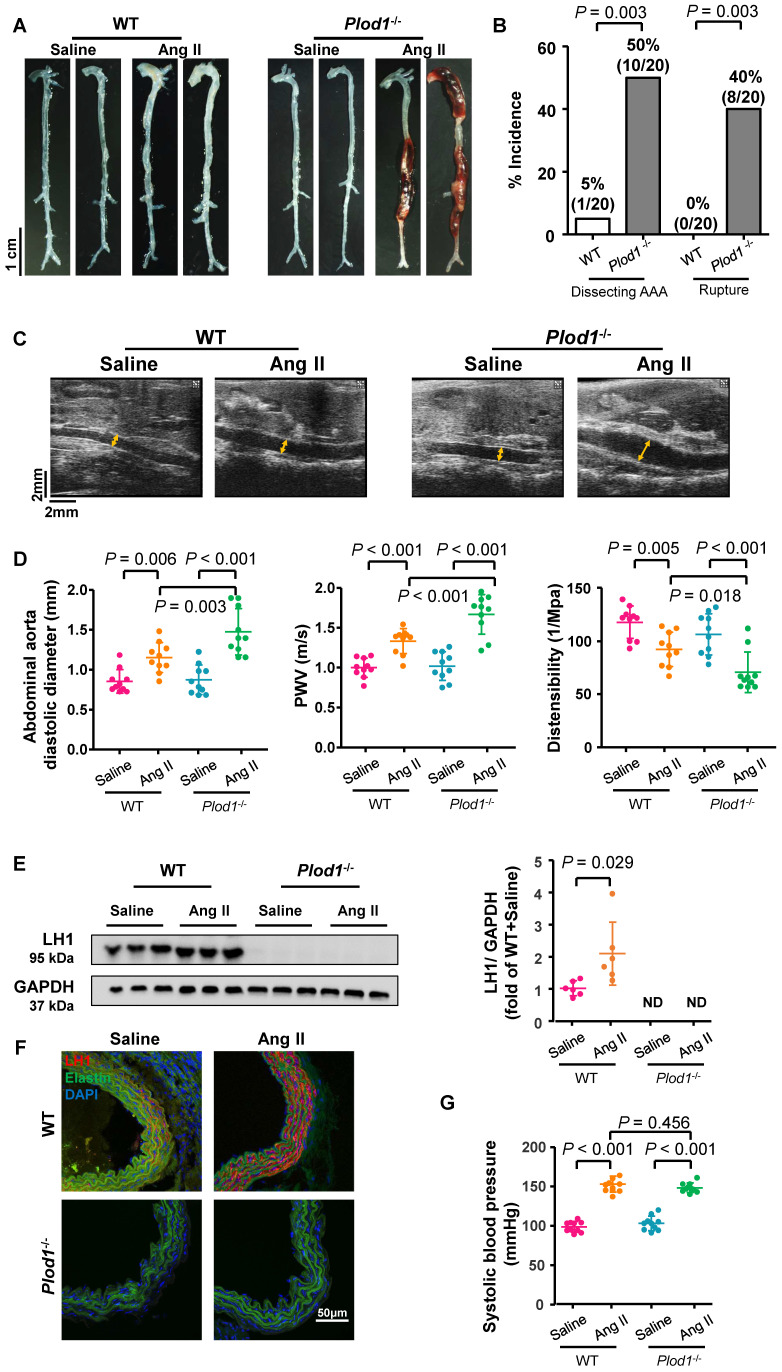
** Lysyl hydroxylase 1 (LH1) deficiency triggers dissecting abdominal aortic aneurysm (AAA) formation following 4 weeks of angiotensin II (Ang II) infusion. A,** Representative morphology of the aortas of wild-type (WT) and procollagen-lysine, 2-oxoglutarate 5-dioxygenase 1 (*Plod1*)*^-/-^* mice. **B,** The incidence of dissecting AAA and aortic aneurysm rupture in WT and *Plod1^-/-^* mice in response to Ang II administration (n = 20). Two-sided fisher's exact test. **C,** Representative images of transabdominal ultrasound measurements (the lumen is indicated by the yellow line) at day 28 after saline or Ang II infusion. **D,** Quantification of the abdominal aorta diastolic diameter, pulse-wave velocity (PWV) and distensibility of the aortic wall by ultrasound at day 28 after saline or Ang II infusion (n = 10). **E,** Representative immunoblotting images of LH1 and quantitative analysis of LH1 expression in the abdominal aortas of WT and *Plod1^-/-^* mice after Ang II or saline infusion (n = 6). ND indicates not detected. Student's unpaired two-tailed t-test. **F,** Representative immunostaining images of LH1 (red), elastin (green) and DAPI (blue) in the abdominal aorta. **G,** The systolic blood pressure of each group at day 28 after saline or Ang II infusion (n = 10). Two-way ANOVA followed by the Bonferroni post hoc test was used in D and G.

To examine the direct role of LH1 in AAA pathogenesis, WT and *Plod1^-/-^* mice were infused with Ang II subcutaneously for 4 weeks to induce dissecting AAA formation. After Ang II infusion, all mice were euthanized, and the whole aortas were collected to evaluate dissecting AAA formation and rupture. No aneurysm was observed in the saline-infused *Plod1^-/-^* mice, suggesting that LH1 deficiency did not result in dissecting AAA development under normal conditions. Macroscopic examination of the aortas revealed that only 5% of the WT mice (1 of 20) developed dissecting AAA in response to Ang II administration, whereas *Plod1^-/-^* mice exhibited significantly aggravated remodeling of the aorta, with 50% of the mice (10 of 20) developing dissecting AAA formation and 40% of the mice (8 of 20) experiencing rupture events (Figure 1A-B). Among these 8 mice with aortic rupture, 75% of the mice (6 of 8) had abdominal aorta rupture events (the presence of hemorrhage of the abdominal aorta between the celiac artery and the left renal artery), and 25% of the mice (2 of 8) had thoracic aortas (the presence of a blood clot in the chest cavity).

Transabdominal ultrasound imaging showed progressively increased diastolic diameter of the suprarenal region of the abdominal aorta of *Plod1^-/-^*mice at days 7, 14 and 28 of Ang II infusion compared with that at baseline (day 0), whereas such an increase was only observed in WT mice at days 14 and 28 infused with Ang II (Figure 1C-D; Figure S1). Notably, the diastolic diameter of the suprarenal region of the abdominal aorta was significantly higher in *Plod1^-/-^* mice than in WT mice at day 7 (1.26 ± 0.29 mm versus 1.03 ± 0.13 mm, *P **=*** 0.019), day 14 (1.40 ± 0.20 mm versus 1.19 ± 0.10 mm, *P **=*** 0.016) and day 28 (1.47 ± 0.29 mm versus 1.15 ± 0.18 mm, *P **=*** 0.003) of Ang II infusion. We further investigated whether an increase in diameter in the abdominal aortas of *Plod1^-/-^* mice was correlated with aortic wall functions. Aortic stiffness as measured by PWV was significantly higher, concomitant with a marked decrease in distensibility in *Plod1^-/-^* compared with WT mice at day 14 (*Plod1^-/-^* versus WT in PWV: 1.38 ± 0.11 m/s versus 1.18 ± 0.11 m/s, *P =* 0.001; *Plod1^-/-^* versus WT in distensibility: 86.88 ± 10.95 1/MPa versus 110.02 ± 6.81 1/MPa, *P <* 0.001) and day 28 (*Plod1^-/-^* versus WT in PWV: 1.68 ± 0.25 m/s versus 1.34 ± 0.16 m/s, *P <* 0.001; *Plod1^-/-^* versus WT in distensibility: 71.52 ± 18.85 1/MPa versus 92.98 ± 16.09 1/MPa, *P =* 0.018) of Ang II infusion (Figure 1D; Figure S1). Furthermore, the protein levels of LH1 in the abdominal aorta were significantly increased upon Ang II infusion (Figure 1E-F), this increase in LH1 expression may represent an important compensatory role that protects against aneurysm development, whereas mice lacking LH1 may have increased susceptibility to dissecting AAA formation and rupture. Amino acid analysis revealed that hydroxylysine levels were significantly increased in WT aorta upon Ang II infusion, whereas mice lacking LH1 had decreased hydroxylysine levels. In contrast, 4-hydroxyproline levels remained unchanged between *Plod1^-/-^* and WT aorta (Figure S2).

Multiple lines of evidence suggest that hypertension is a major independent risk factor for AAA 24. The blood pressure of both *Plod1^-/-^* and WT mice was significantly increased as early as day 7 of Ang II infusion compared with that at the respective baseline, and no further progression was observed at days 14 and 28 (Figure 1G and Figure S3). Importantly, LH1 deficiency did not further affect blood pressure compared with that in WT mice at the respective time points. Overall, these findings provide direct evidence that LH1 deficiency increases susceptibility to dissecting AAA formation and rupture without altering the hypertensive response.

### LH1 deficiency increases structural damage to the aorta in response to Ang II administration

**Figure 2:**
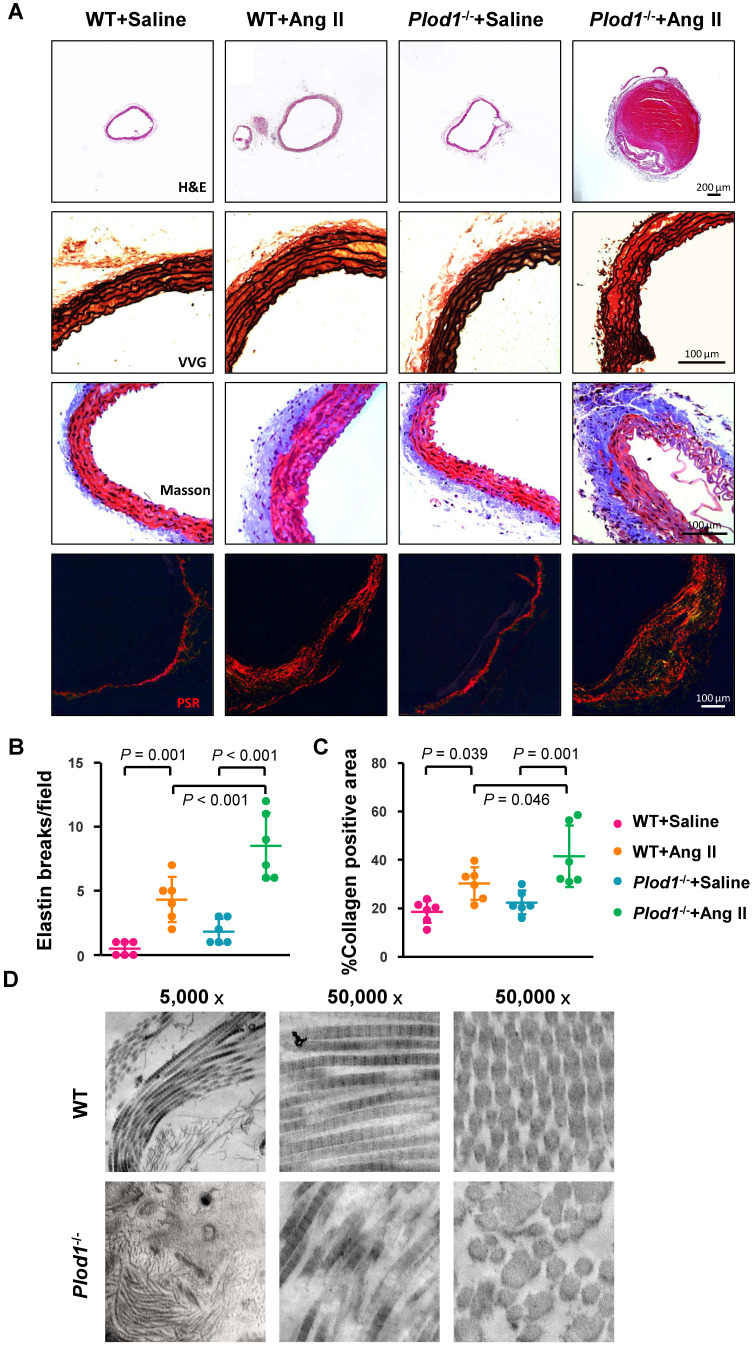
** Lysyl hydroxylase 1 (LH1) deficiency increases structural damage to the aorta in response to angiotensin II (Ang II) administration. A,** Representative cross-sections of hematoxylin and eosin (H&E)-, Verhoeff-Van Gieson (VVG)-, Masson trichrome- and picrosirius red (PSR)-stained abdominal aorta tissues from mice after 4 weeks of Ang II or saline infusion. Quantification of elastin breakage (VVG staining, **B**) and collagen content (PSR staining, **C**) (n = 6). **D,** Transmission electron microscopy images showing an overview, collagen fibers, and cross-sectioned fibers in the abdominal aortas of mice. Two-way ANOVA followed by Bonferroni post hoc test.

Histological analysis of the abdominal aorta with H&E confirmed increased dilatation and showed aortic dissection with a characteristic true lumen and false lumen in the abdominal aortic lesions of *Plod1^-/-^* mice infused with Ang II (Figure 2A). Elastin staining and quantitative analysis demonstrated that LH1 deficiency increased fragmentation of the elastin layer in response to Ang II administration (Figure 2A-B). Moreover, Masson trichrome and PSR staining showed excess adventitial collagen deposition in Ang II-treated *Plod1^-/-^* mice compared with Ang II-treated WT mice (Figure 2A and 2C), indicating that LH1 deficiency promotes aortic fibrosis. To further investigate the collagen ultrastructure in the aortas of *Plod1^-/-^* mice, we performed transmission electron microscopy. We found that the variation in collagen fibril diameter was larger in the aortas of *Plod1^-/-^* mice not infused with Ang II, whereas the diameter was uniform in WT mice. Furthermore, microfibrils with irregular contours were also seen in the samples from *Plod1^-/-^* mice (Figure 2D), indicating that LH1 deficiency led to ultrastructural abnormalities in collagen fibrils. Taken together, these data demonstrated that deficiency of LH1 induces elastin fragmentation and increases aortic fibrosis and defects in the ultra-architecture of collagen in the abdominal aorta, causing the characteristic features of dissecting AAA in response to Ang II administration.

### LH1 deficiency aggravates gene transcription alterations in the abdominal aorta

**Figure 3:**
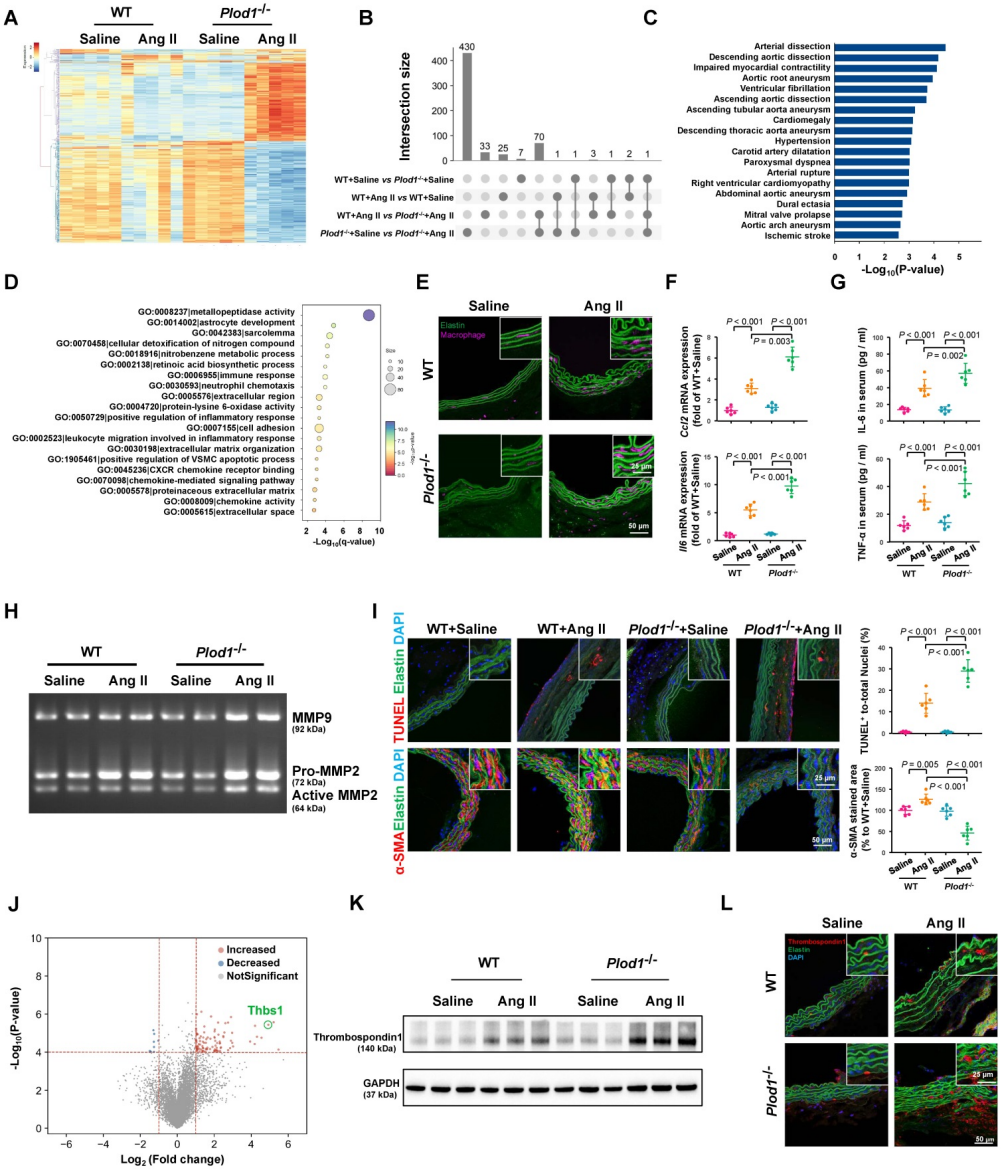
** Lysyl hydroxylase 1 (LH1) deficiency aggravates inflammation, matrix metalloproteinase (MMP) activity and apoptosis of vascular smooth muscle cells (VSMCs) in the abdominal aorta. A,** Cluster analysis heat map showing the transcript levels of differentially expressed genes (DEGs) in the abdominal aortas of wild-type (WT) and procollagen-lysine, 2-oxoglutarate 5-dioxygenase 1 (*Plod1*)*^-/-^* mice following 2 weeks of saline or angiotensin II (Ang II) infusion. The color scale illustrates the relative expression levels across all samples: red represents an expression level above the mean, and blue represents an expression level lower than the mean. The dendrogram on the left of the heat map shows the clustering of the transcripts (n = 5). **B,** UpSet plot of the intersection of four groups. The histogram shows the number of DEGs in each subset. **C,** Human Phenotype Ontology analysis of DEGs between *Plod1*^-/-^ and WT mice after Ang II infusion. **D,** Top enriched terms, as identified by from Gene Ontology (GO) enrichment analysis of the DEGs between *Plod1^-/-^* and WT mice after Ang II infusion. **E,** Representative immunofluorescence staining of macrophages in the abdominal aorta after 4 weeks of saline or Ang II infusion. The insets are higher magnification images. **F,** mRNA expression of the inflammatory cytokines *Ccl2* and *Il6* in the abdominal aorta (n = 6). **G,** Serum levels of the proinflammatory cytokines interleukin (IL)-6 and tumor necrosis factor alpha (TNF-α) were measured by ELISA (n = 6). **H,** Representative gelatin zymography of abdominal aortas from the indicated groups. **I,** Representative images and quantification of apoptotic cells (upper) and α-SMA expression (lower) in the abdominal aorta (n = 6). **J,** Volcano plot displaying DEGs between *Plod1^-/-^* and WT mice after Ang II infusion. **K,** Representative immunoblotting images for thrombospondin-1 in the abdominal aortas of WT and *Plod1^-/-^* mice. **L,** Representative immunostaining images of thrombospondin-1 (red), elastin (green) and DAPI (blue) in the abdominal aorta. Two-way ANOVA followed by Bonferroni post hoc test.

To gain mechanistic insights into the effects of LH1 deficiency on the formation and rupture of dissecting AAA, we performed whole-transcriptomic analysis of abdominal aortic tissue from day 14 after saline or Ang II infusion using RNA sequencing. The sequencing data have been uploaded to the National Center for Biotechnology Information under GEO accession number GSE175683 and are publicly available. In *Plod1^-/-^* mice, a total of 503 genes (209 upregulated and 294 downregulated) were differentially expressed (|logFC| > 1 and q-value < 0.05) between the saline-treated group and the Ang II-treated group. In contrast, in WT mice, only 31 genes (18 upregulated and 13 downregulated) were differentially expressed between these two groups (Figure 3A-B; Table S3 in Supplementary Material). Importantly, we found that a total of 108 genes were differentially expressed between *Plod1^-/-^* and WT mice after Ang II infusion, which might explain the underlying mechanism of LH1 deficiency-induced aggravation of dissecting AAA. By using gProfiler software, the 108 DEGs identified RNA sequencing were analyzed using the Human Phenotype Ontology. Interestingly, enrichment analysis demonstrated that these DEGs were correlated with the pathogenesis of vascular diseases such as arterial dissection, arterial rupture and AAA (Figure 3C), which reinforced our observation that LH1 is involved in dissecting AAA formation. Furthermore, the 108 DEGs were subjected to GO enrichment analysis to identify overrepresented biological functions and canonical pathways. These genes belonged to multiple categories associated with metallopeptidase activity, positive regulation of the inflammatory response, cell adhesion and positive regulation of the vascular associated smooth muscle cell apoptotic process (Figure 3D), which are known pivotal mechanisms of aortic aneurysm formation and rupture 17, 18, 25. KEGG pathway analysis suggested that the DEGs were also enriched in several inflammation-related signaling pathways, such as cytokine-cytokine receptor interactions, the TNF signaling pathway and chemokine signaling (Figure S4). Taken together, the RNA-sequencing data revealed that LH1 deficiency significantly aggravated profound changes in the gene transcriptional profile of the abdominal aorta and that the mechanism of dissecting AAA may involve proinflammatory processes, MMP activation, and VSMC apoptosis.

### The absence of LH1 promotes the proinflammatory process, increases MMP activity, and induces severe VSMC apoptosis in the abdominal aorta

The major characteristic of AAA is the accumulation of infiltrating macrophages accompanied by the expression of proinflammatory cytokines in the aortic wall 17, 25. Immunofluorescence staining revealed markedly greater accumulation of macrophages in the aortas of *Plod1^-/-^* mice than those of WT mice on day 14 after Ang II infusion (Figure 3E; Figure S5). The increase in inflammation in Ang II-treated *Plod1^-/-^* mice was also evidenced by the increased expression levels of *Ccl2* and *Il6* in the aortic wall and increased levels of IL-6 and TNF-α in the serum (Figure 3F-G). Macrophages are thought to be the major sources of MMPs 26, 27 and proteases in the vascular wall that degrade elastin, collagen and laminin, mediating aortic wall remodeling and compromising the structural integrity of the vasculature 28. Thus, we used gelatin zymography to assess MMP activity, which is potentially important for ECM degradation. We found a significantly greater increase in the levels of MMP9, Pro-MMP2 and active MMP2 in the abdominal aortas of Ang II-treated *Plod1^-/-^* mice than those of Ang II-treated WT mice (Figure 3H; Figure S6). It has been reported that MMPs can degrade other non-ECM to mediate apoptosis of VSMCs in the vessel wall 29, which provides structural integrity to the arterial wall. Therefore, we assessed and quantified the extent of VSMC apoptosis. Quantification of TUNEL^+^ cells in the abdominal aorta suggested significantly increased cell apoptosis in Ang II-treated *Plod1^-/-^* mice compared with Ang II-treated WT mice (Figure 3I). Subsequently, the loss of VSMC and a significant reduction in the expression of α-SMA (the main VSMC structural protein) in the abdominal aorta was observed in Ang II-treated *Plod1^-/-^* mice compared with Ang II-treated WT mice (Figure 3I). Taken together, these data demonstrate that loss of LH1 can lead to a proinflammatory processes, increased MMP activity, and severe VSMC apoptosis in the abdominal aorta as suggested by transcriptomic analysis.

### Absence of LH1 significantly upregulates thrombospondin-1 expression in the aneurysmal aorta

To explore the key mediator of dissecting AAA pathogenesis in the aortas of Ang II-treated *Plod1^-/-^* mice, we investigated the contribution of biological processes and signaling pathways that target proinflammatory processes, MMP activity and VSMC apoptosis. As manifested by the volcano plot, we found that *Thbs1* was among the top 10 genes regardless of whether the genes were ranked according to the p-value or LogFC (Figure 3J; Table S3 in Supplementary Material). Thrombospondin-1 is a member of the matricellular thrombospondin protein family. Interestingly, it has been reported that thrombospondin-1 plays a key role in the regulation of macrophage adhesion and the inflammatory response during the pathogenesis of dissecting AAA 26. Moreover, thrombospondin-1 regulates the gelatinase activity of macrophages 30 and induces apoptosis of aortic VSMCs in mouse models of dissecting AAA 17. Immunoblotting and immunofluorescence staining confirmed that the protein levels of thrombospondin-1 were slightly increased in the abdominal aortas of WT mice upon Ang II infusion, while the levels increased to a markedly greater degree in the aortas of Ang II-treated *Plod1^-/-^* mice (Figure 3K-L; Figure S7).

### Thrombospondin-1 is the key mediator of dissecting AAA pathogenesis in the absence of LH1

**Figure 4:**
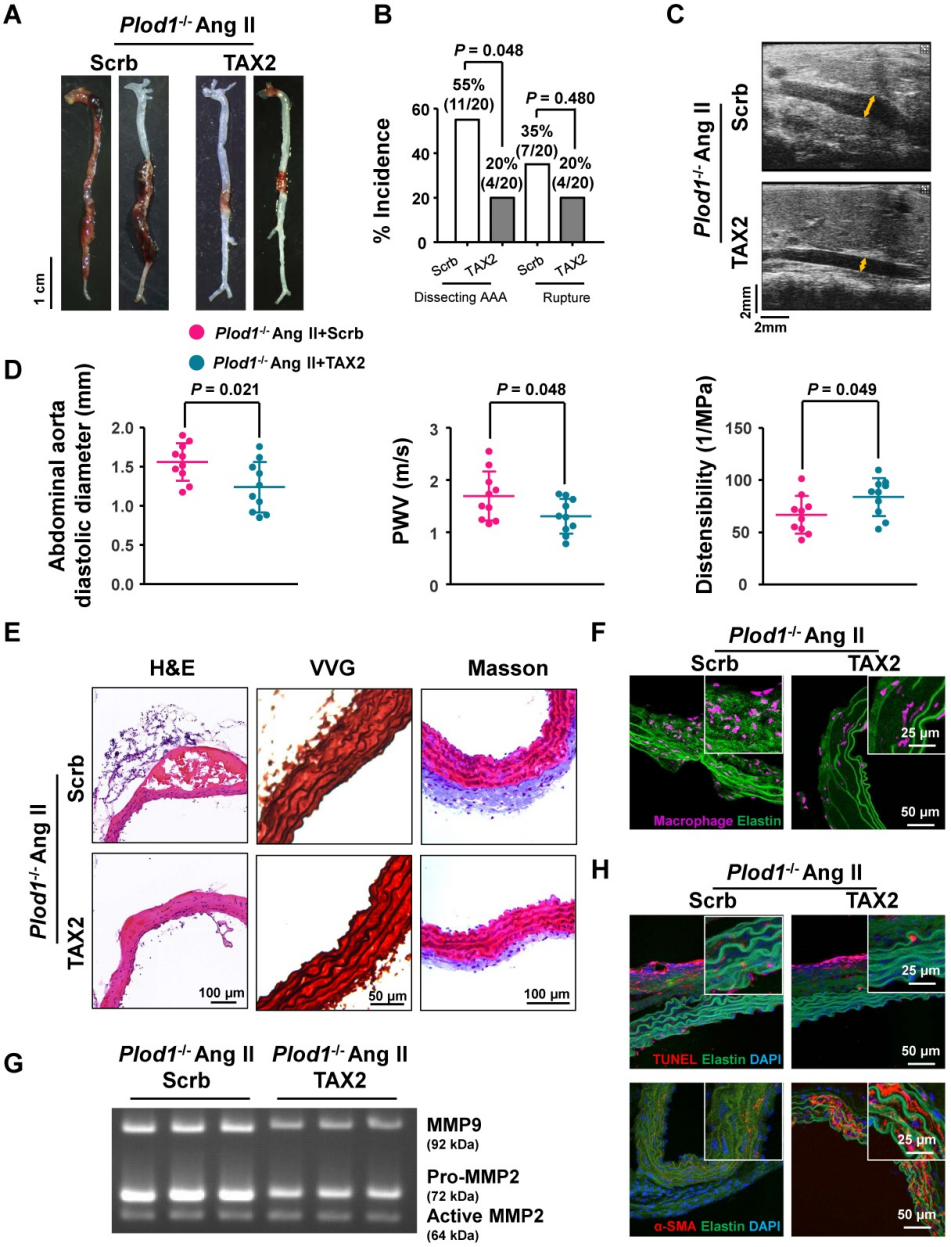
** Thrombospondin-1 is the key mediator of dissecting abdominal aortic aneurysm (AAA) pathogenesis in the absence of lysyl hydroxylase 1 (LH1). A,** Representative aortas from procollagen-lysine, 2-oxoglutarate 5-dioxygenase 1 (*Plod1*)*^-/-^* mice treated with scrambled peptide (scrb) or TAX2 followed by angiotensin II (Ang II) infusion for 4 weeks. **B,** Incidence of dissecting AAA and aortic aneurysm rupture in* Plod1^-/-^* mice treated with scrb or TAX2 in response to Ang II (n = 20). Two-sided fisher's exact test. **C,** Representative images of transabdominal ultrasound measurements (the lumen is indicated by the yellow line). **D,** Quantification of the abdominal aorta diastolic diameter, pulse-wave velocity (PWV) and distensibility of the aortic wall by ultrasound (n = 10). Student's unpaired two-tailed t-test. **E,** Representative hematoxylin and eosin (H&E)-, Verhoeff-Van Gieson (VVG)-, and Masson trichrome-stained cross-sections of the abdominal aortas of mice. **F,** Representative immunofluorescence staining of macrophages in the abdominal aorta following Ang II infusion for 2 weeks. The insets show higher magnification images. **G,** Representative gelatin zymography of abdominal aortas from the indicated groups. **H,** Representative images of apoptotic cells (upper) and α-SMA expression (lower) in the abdominal aorta.

To determine whether thrombospondin-1 is the driving force of dissecting AAA pathogenesis in the absence of LH1, we used TAX2, a CD47-derived cyclic peptide that targets thrombospondin-1, selectively prevents the thrombospondin-1:CD47 interaction and blocks the downstream signaling pathway. At day 28 of Ang II infusion in *Plod1^-/-^* mice, macroscopic examination of the aortas demonstrated that 55% of mice (11 of 20) showed dissecting AAA formation and 35% of the mice (7 of 20) experienced rupture events when treated with scrambled peptide, whereas TAX2 treatment exhibited strong inhibition of the incidence of the formation of dissecting AAA (4 of 20) (Figure 4A-B). The diastolic diameter of the abdominal aorta and aortic stiffness, as measured by transabdominal ultrasound imaging, were also significantly attenuated in TAX2-treated* Plod1^-/-^* mice compared with scrambled peptide-treated* Plod1^-/-^* mice after Ang II administration (TAX2-treated versus scrambled peptide-treated in diameter: 1.24 ± 0.32 mm versus 1.56 ± 0.24 mm, *P =* 0.021; TAX2-treated versus scrambled peptide-treated in PWV: 1.31 ± 0.33 m/s versus 1.70 ± 0.47 m/s, *P =* 0.048; TAX2-treated versus scrambled peptide-treated in distensibility: 83.8 ± 18.12 1/Mpa versus 66.72 ± 18.04 1/Mpa, *P =* 0.049; Figure 4C-D). Furthermore, histological analysis of the aortic tissues revealed decreased elastin fragmentation and adventitial collagen deposition after inhibition of thrombospondin-1 (Figure 4E; Figure S8). As anticipated, TAX2 treatment blunted the accumulation of macrophages (Figure 4F; Figure S9) and suppressed MMP activity (Figure 4G; Figure S10) and VSMC apoptosis (Figure 4H; Figure S11) in the aortas of *Plod1^-/-^* mice. Taken together, these data support the notion that dissecting AAA pathogenesis in the absence of LH1 may primarily be mediated by thrombospondin-1.

### Correction of LH1 deficiency by AAV-based gene therapy prevents Ang II-induced dissecting AAA formation in *Plod1^-/-^* mice

**Figure 5:**
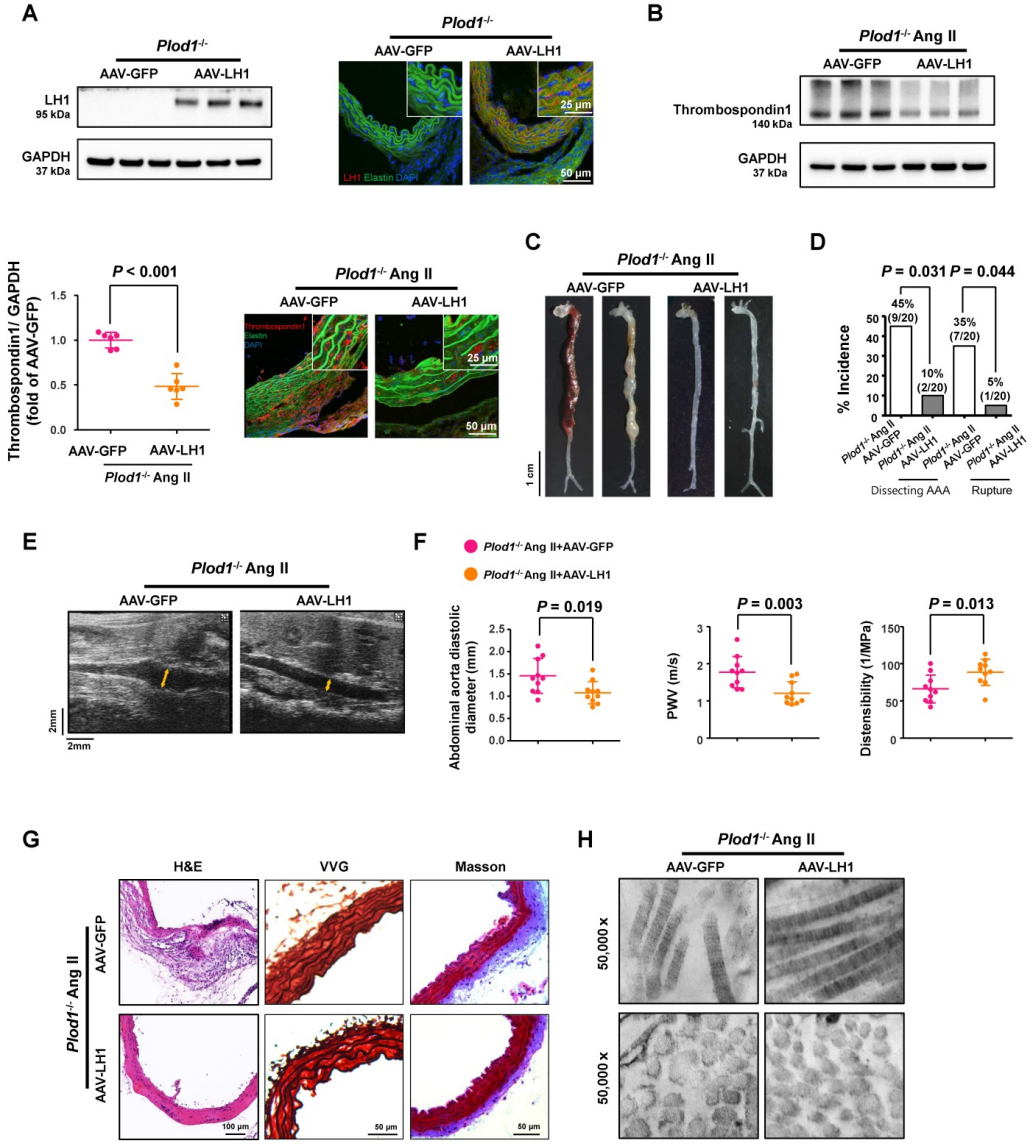
** Correction of lysyl hydroxylase 1 (LH1) deficiency by adeno-associated virus (AAV)-based gene therapy prevents angiotensin II (Ang II)-induced dissecting abdominal aortic aneurysm (AAA) formation in procollagen-lysine, 2-oxoglutarate 5-dioxygenase 1 (*Plod1*)^-/-^ mice. A,** Representative immunoblot analysis and immunofluorescence images of LH1 expression in the abdominal aortas of *Plod1*^-/-^ mice treated with AAV-GFP or AAV-LH1. The insets show higher magnification images. **B,** Representative immunoblot analysis and immunofluorescent images of thrombospondin-1 expression in the abdominal aortas of *Plod1*^-/-^ mice treated with AAV-GFP or AAV-LH1 followed by Ang II infusion for 2 weeks (n = 6). Student's unpaired two-tailed t-test. **C,** Representative aortas from* Plod1^-/-^* mice treated with AAV-GFP or AAV-LH1 followed by Ang II infusion for 4 weeks. **D,** Incidence of dissecting AAA and aortic aneurysm rupture in response to Ang II in* Plod1^-/-^* mice treated with AAV-GFP or AAV-LH1 (n = 20). Two-sided fisher's exact test. **E,** Representative images of transabdominal ultrasound measurements (the lumen is indicated by the yellow line). **F,** Quantification of the abdominal aorta diastolic diameter, pulse-wave velocity (PWV) and distensibility of the aortic wall by ultrasound (n = 10). Student's unpaired two-tailed t-test. **G,** Representative hematoxylin and eosin (H&E)-, Verhoeff-Van Gieson (VVG)-, and Masson trichrome-stained cross-sections of the abdominal aortas of mice. H, Transmission electron microscopy images showing collagen fibers and cross-sectioned fibers in the abdominal aortas of mice.

To further clarify whether LH1 plays a causative role in preventing dissecting AAA formation and to study the potential effect of gene therapy in rescuing the expression of LH1, *Plod1^-/-^* mice were intraperitoneally injected with 2×10^11^ AAV-LH1 or AAV-GFP (as an experimental negative control) in a total volume of 200 µl of supernatant. At 3 weeks after transfection, we first examined the expression of LH1. As expected, the protein expression of LH1, as assessed by immunoblotting and immunofluorescence staining, was significantly rescued in the abdominal aortas of *Plod1^-/-^* mice treated with AAV-LH1 but not those of *Plod1^-/ -^* mice treated with AAV-GFP (Figure 5A). We also measured the protein level of thrombospondin-1 at day 14 of Ang II infusion, which was markedly decreased in AAV-LH1-treated mice compared to AAV-GFP-treated mice (Figure 5B), indicating that LH1 might play a possible role as a negative regulator of thrombospondin-1 expression in the abdominal aorta. Four weeks after Ang II infusion, macroscopic examination of the aortas demonstrated that 45% of mice (9 of 20) showed dissecting AAA formation and 35% of the mice (7 of 20) experienced rupture events when treated with AAV-GFP, whereas AAV-LH1 treatment exhibited strong inhibition of the incidence of the formation of dissecting AAA (2 of 20) and rupture (1 of 20) (Figure 5C-D). The diastolic diameter of the abdominal aorta and aortic stiffness were also markedly attenuated in AAV-LH1-treated mice compared with AAV-GFP-treated mice (AAV-LH1-treated versus AAV-GFP-treated in diameter: 1.08 ± 0.25 mm versus 1.46 ± 0.39 mm, *P =* 0.019; AAV-LH1-treated versus AAV-GFP-treated in PWV: 1.21 ± 0.31 m/s versus 1.77 ± 0.42 m/s, *P =* 0.003; AAV-LH1-treated versus AAV-GFP-treated in distensibility: 88.70 ± 17.59 1/Mpa versus 66.28 ± 18.71 1/Mpa, *P =* 0.013; Figure 5E-F). Furthermore, histological analysis of the aortic tissues revealed decreased elastin fragmentation and adventitial collagen deposition (Figure 5G; Figure S12) and normalization of ultrastructural abnormalities in collagen fibrils (Figure 5H) after correction of LH1 deficiency. Taken together, these findings indicate that correction of LH1 expression in *Plod1^-/ -^* mice by AAV-based gene therapy substantially alleviated Ang II-induced dissecting AAA formation and preserved aortic structure and function.

### LH1 are significantly reduced and thrombospondin-1 levels are elevated in specimens from humans with AAA

**Figure 6:**
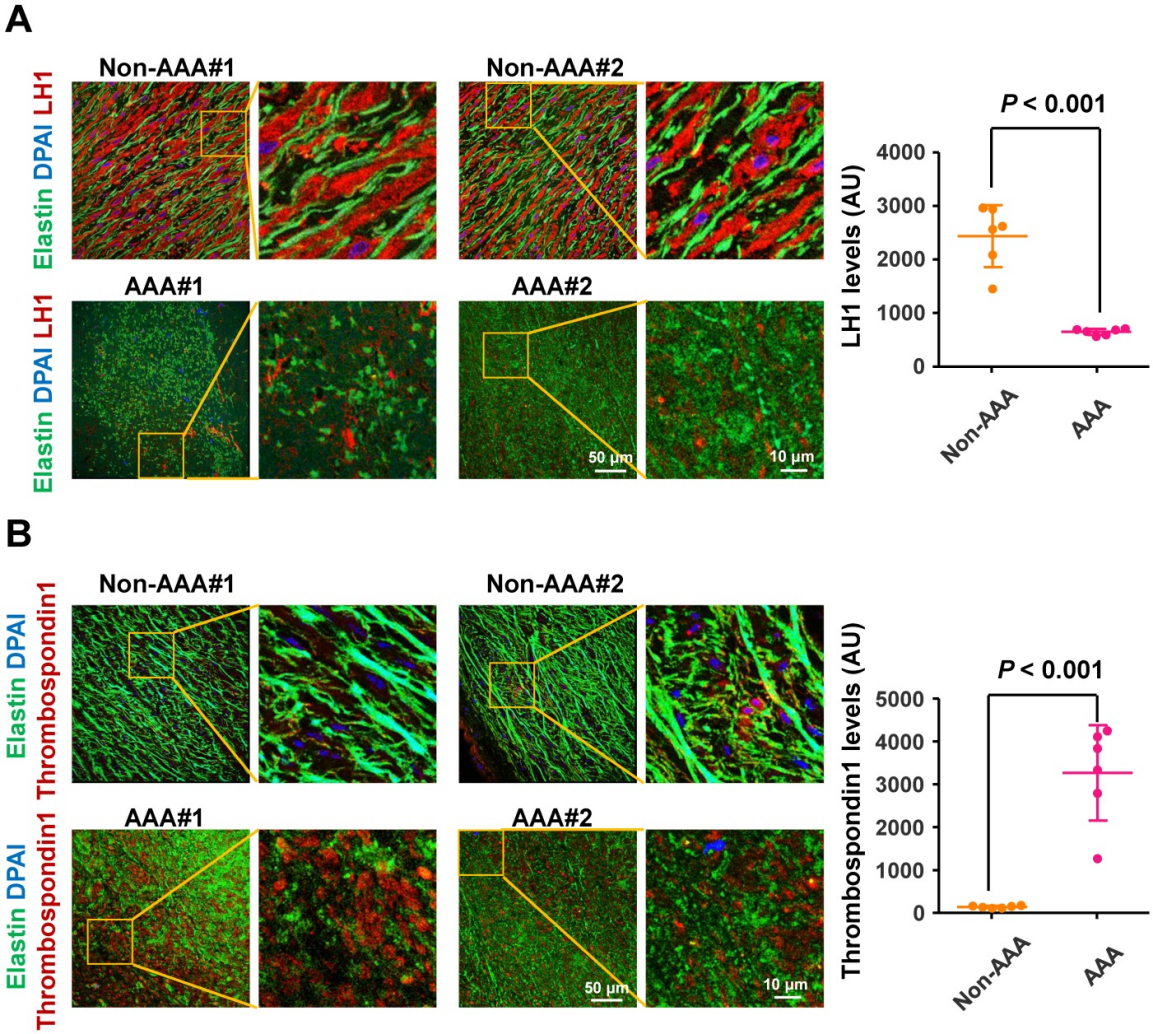
** Lysyl hydroxylase 1 (LH1) are significantly reduced and thrombospondin-1 levels are elevated in specimens from humans with abdominal aortic aneurysm (AAA).** Representative immunofluorescence images and quantification of LH1 (**A**) and thrombospondin-1 (**B**) expression. The boxed regions in the images are shown at a higher magnification on the right (n = 6). AU indicates arbitrary units. Student's unpaired two-tailed t-test.

To determine the changes in LH1 and thrombospondin-1 expression in the aortas of human AAA, we collected abdominal aortic specimens from 6 patients with AAA (abdominal aortas > 40 mm diameter) undergoing open aortic repair and aortic specimens from 6 control subjects who underwent heart transplantation for hypertrophic cardiomyopathy or dilated cardiomyopathy but did not have AAA (abdominal aortas < 30 mm diameter). Immunofluorescence staining revealed fragmented elastin fibers markedly reduced LH1 expression (Figure 6A) and elevated thrombospondin-1 levels (Figure 6B) in specimens from patients with AAA compared with specimens from control subjects. These observations are consistent with the changes we observed in the* Plod1^-/ -^*mouse model of AAA in the present study, supporting the link between deficiency of LH1 and the subsequent increase in thrombospondin-1 expression as a contributing mechanism to AAA formation.

## Discussion

To date, little is known about the pathogenesis of AAA, and there are few available pharmacological options for the prevention and treatment of AAA. The present study provides the first evidence of the direct role of LH1 in the pathogenesis of Ang II-induced dissecting AAA using *Plod1^-/ -^* mice as a model. We found that Ang II increased LH1 levels in the abdominal aortas of WT mice. However, mice lacking LH1 developed dissecting AAA following 4 weeks of Ang II infusion. Similarly, AAA specimens from patients exhibited a marked reduction in LH1 levels in the abdominal aortic wall. Therefore, we speculate that LH1 could exert a protective effect against dissecting AAA formation since LH1 loss or a decrease in its expression is associated with dissecting AAA. To gain mechanistic insights into the effects of the absence of LH1 on the formation of dissecting AAA, we performed whole-transcriptomic analysis, which demonstrated that LH1 deficiency aggravated gene transcription alterations; in particular, *Thbs1* expression was markedly upregulated in the aortas of Ang II-treated *Plod1^-/-^*mice. We then identified increases in proinflammatory processes, an increase in MMP activity and severe VSMC apoptosis, which were mediated predominately by thrombospondin-1, as the primary mechanisms underlying dissecting AAA in* Plod1^-/-^* mice. Moreover, we corrected LH1 expression in *Plod1^-/ -^*mice and observed substantial alleviation of Ang II-induced dissecting AAA formation and preservation of aortic structure and function.

Several vascular diseases, including AAA, are characterized by collagen fibril abnormalities in the vessel wall 31. A recent study reported that abnormal collagen fibrils accompanied by dysregulated collagen fibrillogenesis, including compromised D periodicity and increased fibril curvature, were present in the vascular tissue in both AAA patients and Ang II-induced murine models 8. It is interesting to note that collagen fibrillogenesis is mainly modulated by crosslinking enzymes, which mediate posttranslational modifications and provide stability and connectivity of collagen fibrils 32. One of the key crosslinking enzymes of collagen is LH1, which converts triple helical lysyl residues to hydroxylysyl and mediates cross-link formation and collagen fibrillogenesis and maturation 33. Mutations in the *PLOD1* gene result in LH1 deficiency and the clinical phenotype of the kyphoscoliotic subtype of EDS, a syndrome associated with severe aortopathy including aortic dilation and dissection 34. However, EDS is a rare autosomal recessive connective tissue disorder that is associated with AAA. Various environmental factors, such as trauma, acute or chronic infection and inflammatory diseases, rather than genetic factors are the main causes of AAA 2. Thus, in the present study, we excluded AAA patients with rare heritable disorders such as Marfan syndrome, Loeys-Dietz syndrome or EDS to elucidate the role of LH1 in the pathogenesis of AAA more generally. We found that AAA specimens from patients exhibited a marked reduction in LH1 levels in the abdominal aortic wall (Figure 6). These observations are consistent with the changes we observed in the *Plod1^-/ -^*mouse model of dissecting AAA, suggesting that deficiency of LH1 is a potential contributing mechanism to dissecting AAA formation. AAA was once traditionally regarded as a consequence of arteriosclerosis 35. However, recent studies have indicated that aneurysms arise through pathogenic mechanisms that differ from those responsible for athero-occlusive disease 36, 37. Atherosclerosis may not be a causal event in AAA but develops in parallel with or secondary to aneurysmal dilatation 38. Moreover, an increased pyridinoline:deoxypyridinoline ratio (an indicator of LH-1 activity) has been found in atherosclerotic plaques, which indicates that LH-1 may be relevant to the mechanisms underlying vulnerability and rupture of plaques 39. Thus, to exclude arteriosclerosis as a potential confounder when evaluating the role of LH1 in aneurysm pathogenesis, only abdominal aorta specimens from patients with AAA but not atherosclerosis were used in our study. Similarly, we used C57Bl/6NCrl mice instead of the well-known Apoe^-/-^ mouse model of dissecting AAA because Apoe^-/-^ mice have been shown to have atherosclerotic plaques even if fed a standard laboratory diet 25. Our data demonstrate a direct role for LH1 in the pathogenesis of dissecting AAA independent of arteriosclerosis.

Using TEM, we found profound variation in microfibril diameter and found microfibrils with irregular contours in the aortas of *Plod1^-/-^* mice in the absence of Ang II infusion (Figure 2D). However, no aneurysm (Figure 1A-B) or structural damage to the aorta (Figure 2A) was observed in *Plod1^-/-^* mice under normal conditions. Similar to our results, another study also observed that the aortic wall appears normal under light microscopy in samples collected from sacrificed mice, but ultrastructural analysis revealed degenerated smooth muscle cells and abnormal collagen fibrils in the aortas of *Plod1^-/-^* mice 3. These data indicate that the ultrastructural abnormalities in the aortas of *Plod1^-/-^* mice do not directly lead to the formation of dissecting AAA and that Ang II infusion is a key predisposing factor. Hypertension is as a pivotal comorbidity of AAA development. In the present study, blood pressure measurements showed a significant but comparable hypertensive response in WT and *Plod1^-/-^* mice after Ang II infusion, indicating that an antihypertension-independent mechanism was involved in the protective effects of LH1 against the pathogenesis of dissecting AAA.

How LH1 deficiency promotes dissecting AAA pathogenesis remains elusive. We performed nonbiased RNA sequencing and bioinformatics analysis to investigate the underlying mechanisms. We found that loss of LH1 led to a proinflammatory process, increased MMP activity, and severe VSMC apoptosis in the abdominal aorta, which have all been demonstrated to contribute to AAA development 37, 40. Significant enrichment of these pathophysiological processes in LH1-deficient mice was further validated by immunofluorescence staining and gelatin zymography. Our study is in line with a previous studying involving mRNA microarray analysis of the aortas of a well-established Ang II-induced Apoe^-/-^ mouse model, which showed that the expression of genes encoding inflammatory cytokines (such as CCL2, CXCR4, TNF-α and IL-6), macrophage activation markers (such as CD68) and metalloproteases (such as MMP-9, MMP-12, MMP-19 and ADAM metallopeptidase) was significantly upregulated. Moreover, those DEGs were correlated with the pathogenesis of aortic dissection and EDS 25. However, in the present study, the absence of EDS was confirmed by our enrichment analysis based on the Human Phenotype Ontology, indicating that the pathogenesis of aneurysms in LH1-deficient mice, at least in part, from that underlying vascular incidents in EDS patients and that loss of LH1 function may not be the only underlying mechanism responsible for aneurysm formation in EDS patients.

Thrombospondin-1 is a member of the matricellular thrombospondin protein family and naturally exists as a homotrimeric glycoprotein. The absence of LH1 resulted in highly elevated *Thbs1* levels in the aortas of *Plod1^-/-^* mice. The marked rise in THBS1 levels was concomitant with pathophysiological changes, including inflammation, MMP activation and VSMC apoptosis, in the abdominal aorta. Infiltration of inflammatory cells is a key pathological event that has received substantial attention in aneurysm research. A recent study showed that thrombospondin-1 plays a key role in the regulation of macrophage adhesion and recruitment in the inflammatory response during the pathogenesis of dissecting AAA by using *Thbs1*^-/-^ mice 26. Elevated MMP protein expression was found in human aneurysmal tissues and were reported to correlate with aneurysm development 41, 42. However, in a modified AAA mouse model induced by CaCl_2_, a lack of thrombospondin-1 was shown to inhibit aneurysm formation by elevating tissue inhibitor of metalloproteinases-1 (TIMP1) expression but without affecting MMP expression 30. We speculate that it is possible that the inconsistency in MMP activity is caused by different ECM environments that are likely to be differentially altered in different aneurysm models. VSMCs are pivotal for renewal of aortic ECM proteins and responsible for aortic contractile function. VSMC apoptosis-induced impaired aortic contractility is the key contributor to aneurysm formation 43. A previous report showed that recombinant thrombospondin-1 alone is sufficient to induce apoptosis of aortic SMCs, confirming the key role of thrombospondin-1 in this process 17. Thrombospondin-1 has been reported to mediate apoptosis of VSMCs through its association with CD47 44. In our study, we used TAX2 to selectively prevent the thrombospondin-1:CD47 interaction and subsequently decreased VSMC apoptosis in the aortas of *Plod1^-/-^* mice. Notably, Krishna et al reported the opposite finding: that a high serum thrombospondin-1 concentration was associated with slower AAA growth and that targeting thrombospondin-1 promoted AAA progression in mice 45, 46. These controversial findings reflect the complexity of AAA pathophysiology and the potential multiple functions of thrombospondin-1. However, in the present study, targeting thrombospondin-1 with TAX2 was shown to strongly inhibit dissecting AAA formation in *Plod1^-/-^* mice, supporting the notion that dissecting AAA pathogenesis in the absence of LH1 may be predominately mediated by thrombospondin-1.

AAV-mediated gene therapy, which exerts long-term therapeutic effects, is currently approved for the treatment of several diseases and has been used in preclinical studies and clinical trials 47. Zhao et al reported that *in vivo* AAV-mediated low-density lipoprotein receptor (*Ldlr*) gene correction, which can partially rescue *Ldlr* expression, effectively ameliorates atherosclerosis phenotypes in a *Ldlr* mutant mouse model 48. In our mouse study, partial restoration of the LH1 protein expression in the aorta and partial normalization of thrombospondin-1 expression were observed after *Plod1^-/-^* mice were intraperitoneally injected with AAV-LH1 *in vivo*. These changes correlated with marked normalization of structural, functional and biochemical abnormalities in the aorta, suggesting that although the AAV-mediated gene therapy strategy used to correct LH1 expression in this study was not sufficient to completely alleviate dissecting AAA, gene therapy could be combined with conventional interventions to provide a potential better therapeutic strategy for AAA patients.

We acknowledge that there are limitations to our study. The aortic root that we used may not be an appropriate control sample for the study because thoracic aortas and abdominal aortas may have different protein amounts and distributions (such as LH1) due to variations in structure, hemodynamic forces, extracellular matrix composition, smooth muscle cell phenotype and pathological genetics 49. Control specimens from the abdominal aorta would be ideal. Moreover, the linkage between LH1 and thrombospondin-1 is not well described in the current study. Impaired cross-linking affects the tensile strength of collagen and its role in maintaining mechanical homeostasis and vascular integrity 3, 50, 51. Notably, recent studies by Yamashiro et al. demonstrated that maladaptive upregulation of thrombospondin-1 by mechanical stretch is a driver of thoracic aortic aneurysm in mice 52, 53. Thus, we speculate that impaired cross-linking of collagen induced by LH1 deficiency may trigger abnormal mechanosensing or mechanical stretch, which leads to upregulation of thrombospondin-1 expression in the abdominal aorta of mice. Further studies are clearly warranted to elucidate the underlying mechanism by which LH1 regulates thrombospondin-1 in dissecting AAA.

In conclusion, we demonstrated that LH1 is a pivotal regulator of dissecting AAA pathophysiology. Deficiency of LH1, along with a subsequent increase in thrombospondin-1 expression, triggers an increase in the proinflammatory process, and increase in MMP activity and severe VSMC apoptosis, ultimately leading to dissecting AAA. *In vivo* LH1 expression correction by AAV-mediated gene therapy effectively attenuates susceptibility to dissecting AAA. Notably, we provide novel insight into the critical role of LH1 in the molecular mechanism of dissecting AAA to aid the discovery effective therapeutic approaches to prevent disease progression.

## Supplementary Material

Supplementary figures and tables 1-2.

Supplementary table 3.

## Abbreviations

AAA: abdominal aortic aneurysm
LH: lysyl hydroxylase
Ang II: angiotensin II
ECM: extracellular matrix
WT: wild-type
MMP: matrix metalloproteinases
VSMCs: vascular smooth muscle cells
PLOD1: procollagen-lysine, 2-oxoglutarate 5-dioxygenase 1
EDS: Ehlers-Danlos syndrome
AAV: adeno-associated viral
PWV: pulse-wave velocity
DEGs: differentially expressed genes
GO: gene ontology
IL: interleukin
TNF-α: tumor necrosis factor alpha
SBP: systolic blood pressure
DBP: diastolic blood pressure
IMT: intima-media thickness
