# Supplementary material for: Lysyl hydroxylase 1 (LH1) deficiency promotes angiotensin II (Ang II)-induced dissecting abdominal aortic aneurysm
Source: Theranostics. 2021 Sep 21;11(19):9587–604. doi: 10.7150/thno.65277 (PMC8490513; doi:10.7150/thno.65277)
Supplement: Supplementary file 1 — Supplementary figures and tables 1-2. [file thnov11p9587s1.pdf]

Supplementary Material

Supplementary Figures

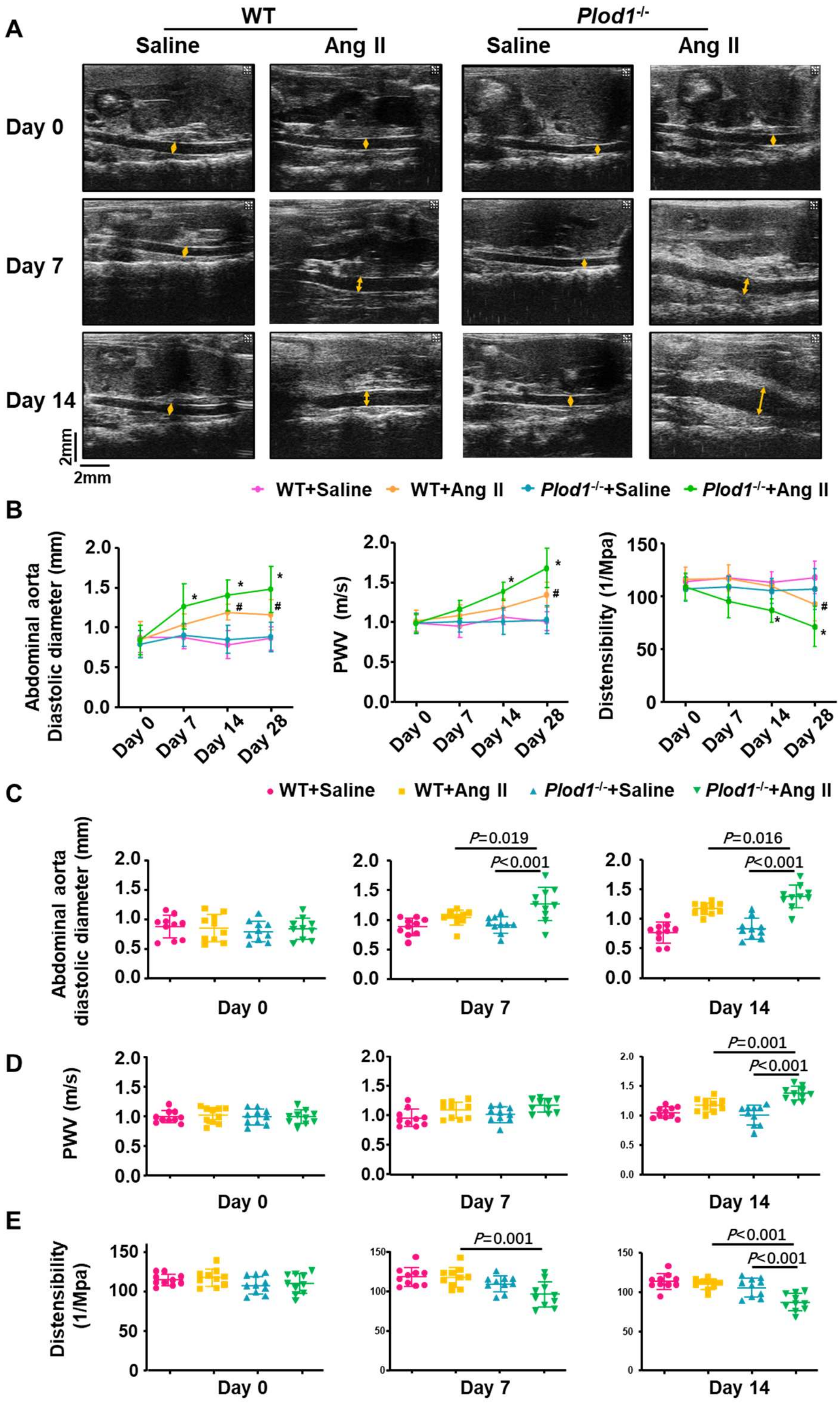

Figure S1 The changes in abdominal aortic structure and function at different time points after saline or angiotensin II (Ang II) infusion in mice. A,

Representative images of transabdominal ultrasound measurements (lumen is indicated in yellow line) at different time points after saline or Ang II infusion from wild-type (WT) and *Plod1*$^{-/-}$ mice. **B,** Quantification of abdominal aorta diastolic diameter, pulse-wave velocity (PWV) and distensibility of aortic wall by ultrasound at different time points after saline or Ang II infusion (n = 10). **P* < 0.05 vs corresponding baseline (day 0) levels of *Plod1*$^{-/-}$ group, #*P* < 0.05 vs corresponding baseline levels of WT group. One-way ANOVA with the Bonferroni post hoc test. Quantification of abdominal aorta diastolic diameter (**C**), PWV (**D**) and distensibility (**E**) of aortic wall by ultrasound at baseline, day 7 and day 14 after saline or Ang II infusion (n = 10). Two-way ANOVA with the Bonferroni post hoc test.

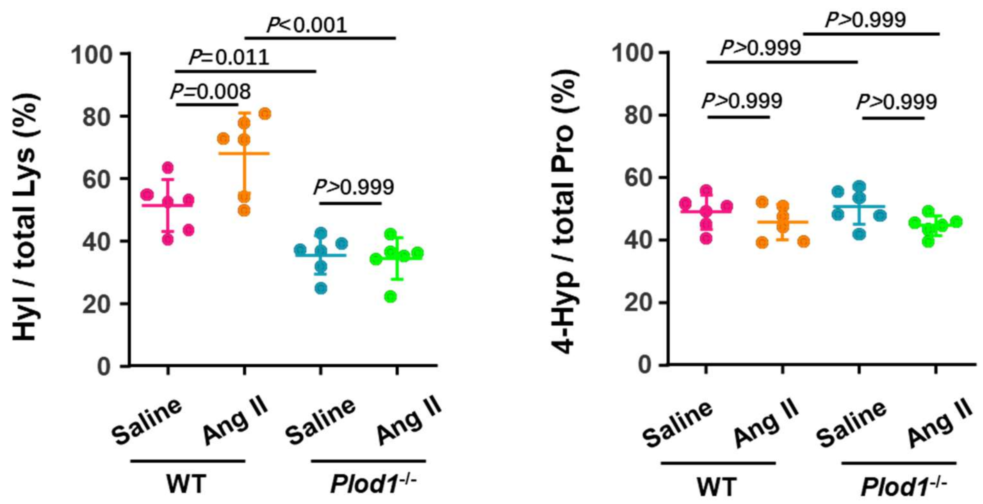

Figure S2 Comparison of lysine and proline posttranslational modifications in wild-type (WT) and *Plod1*$^{-/-}$ aortas after saline or angiotensin II (Ang II) infusion. Quantification of unmodified lysine (Lys), unmodified proline (Pro), hydroxylysine (Hyl), and 4-hydroxyproline (4-Hyp) was performed by UPLC-MS/MS assay in WT and *Plod1*$^{-/-}$ aortas. The percentage was calculated as the concentrations of hydroxylated lysine/proline divided by the concentrations of total lysine/proline (n = 6). Two-way ANOVA with the Bonferroni post hoc test.

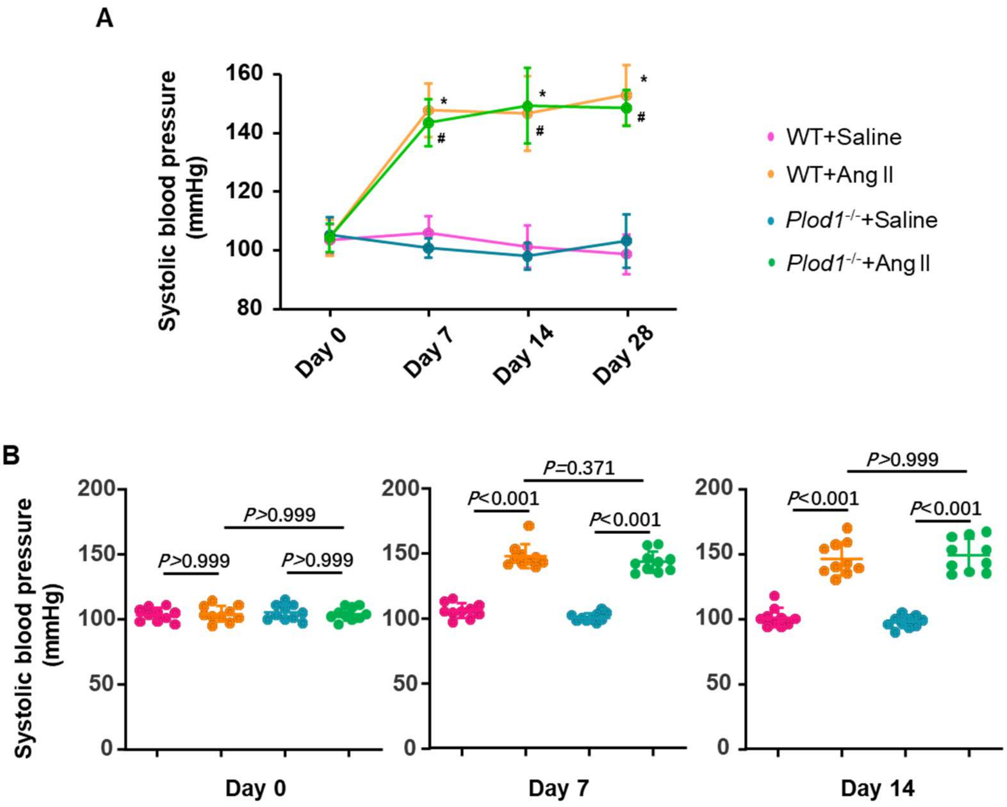

Figure S3 The changes in systolic blood pressure at different time points after saline or angiotensin II (Ang II) infusion in mice. A, Quantification of systolic blood pressure at different time points after saline or Ang II infusion from wild-type (WT) and *Plod1*$^{-/-}$ mice (n = 10). **P* < 0.05 vs corresponding baseline (day 0) levels of *Plod1*$^{-/-}$ group, #*P* < 0.05 vs corresponding baseline levels of WT group. One-way ANOVA with the Bonferroni post hoc test. **B,** Quantification of systolic blood pressure at baseline, day 7 and day 14 after saline or Ang II infusion (n = 10). Two-way ANOVA with the Bonferroni post hoc test.

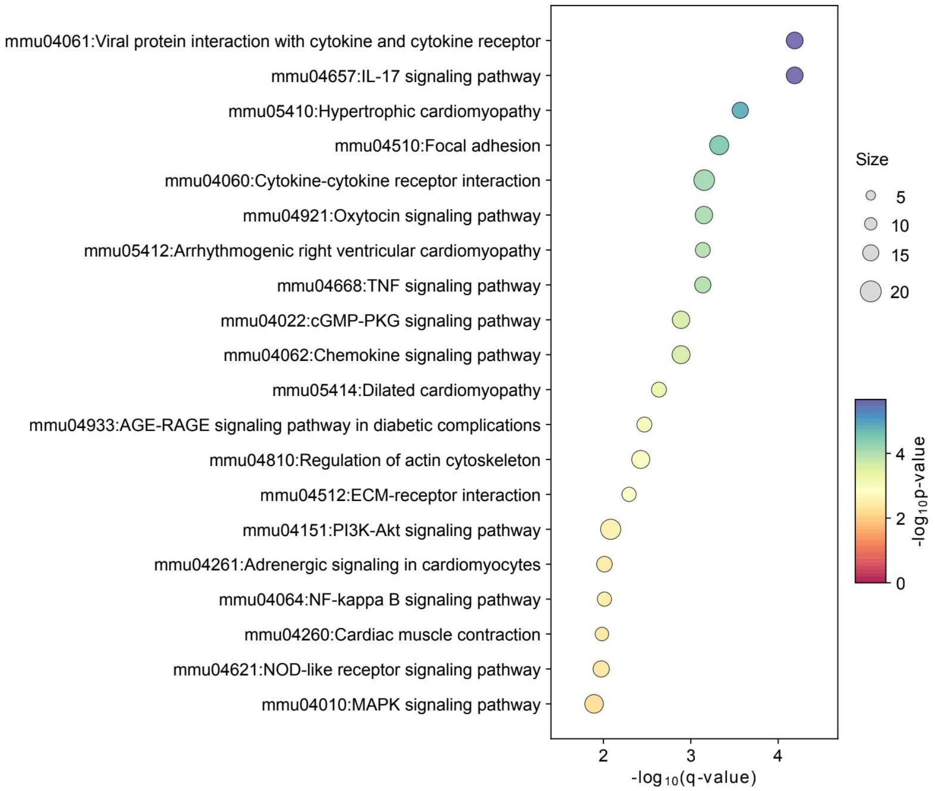

Figure S4. Top terms showing enrichment from KEGG pathways analysis of the genes that are differentially expressed between *Plod1*$^{-/-}$ and wild-type mice after angiotensin II infusion.

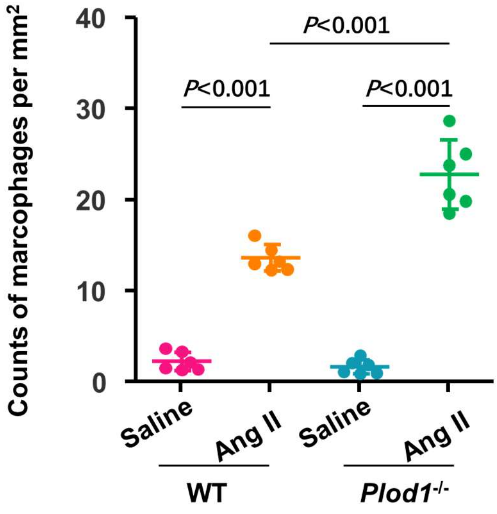

Figure S5. Quantification of macrophage population in abdominal aortas from wild-type (WT) and *Plod1*$^{-/-}$ mice following 2 weeks of saline or angiotensin II (Ang II) infusion. n = 6. Two-way ANOVA with the Bonferroni post hoc test.

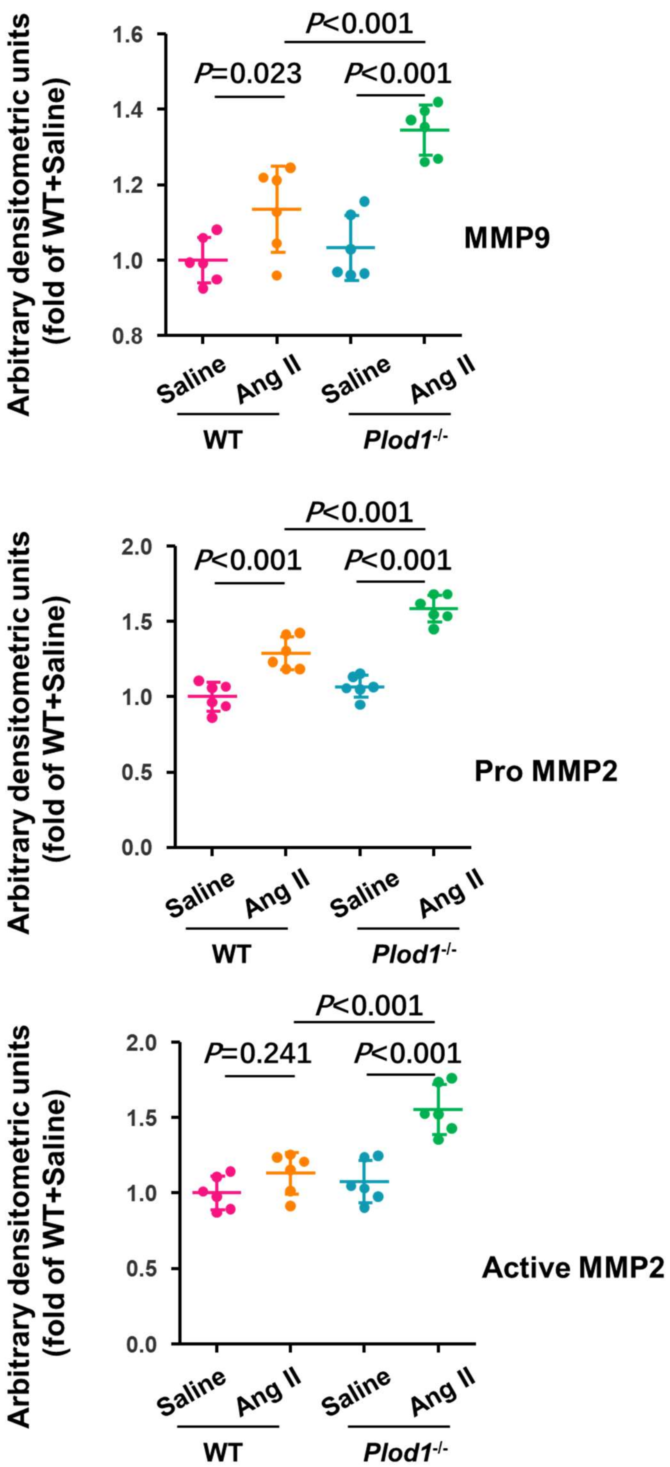

Figure S6. Quantitative analysis of the MMP9, Pro-MMP2 and MMP2 activity in abdominal aortas from wild-type (WT) and *Plod1*$^{-/-}$ mice following 2 weeks of saline or angiotensin II (Ang II) infusion. n = 6. Two-way ANOVA with the Bonferroni post hoc test.

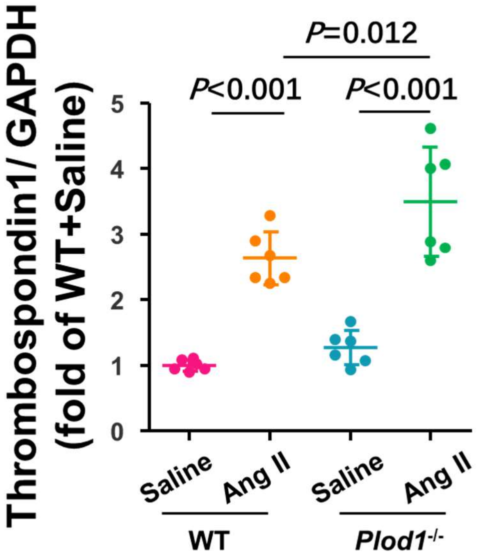

Figure S7. Quantitative analysis of immunoblot for thrombospondin-1 in the abdominal aorta from wild-type (WT) and *Plod1*$^{-/-}$ mice after angiotensin II (Ang II) or saline infusion. n = 6. Two-way ANOVA with the Bonferroni post hoc test.

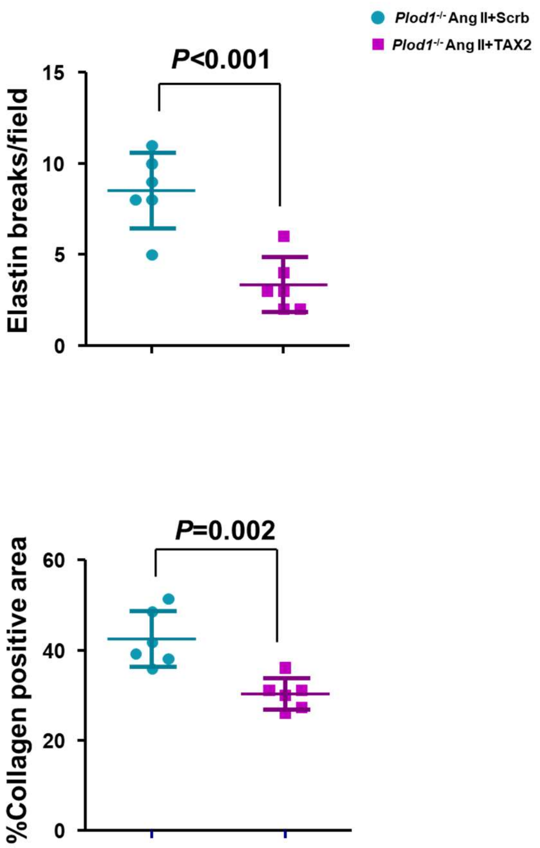

Figure S8. Quantification of elastin breakage (upper) and collagen content (lower) in abdominal aortas from *Plod1*$^{-/-}$ mice treated with scrambled peptide (Scrb) or TAX2 followed by angiotensin II (Ang II) infusion for 2 weeks. n = 6. Student's unpaired two-tailed t-test.

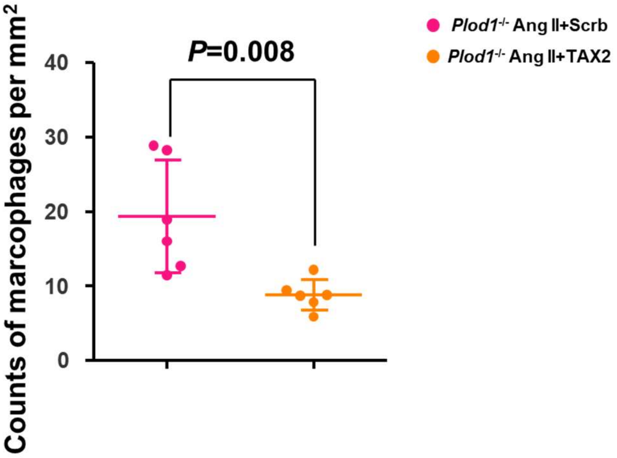

Figure S9. Quantification of macrophage population in abdominal aortas from *Plod1*$^{-/-}$ mice treated with scrambled peptide (Scrb) or TAX2 followed by angiotensin II (Ang II) infusion for 2 weeks. n = 6. Student's unpaired two-tailed t-test.

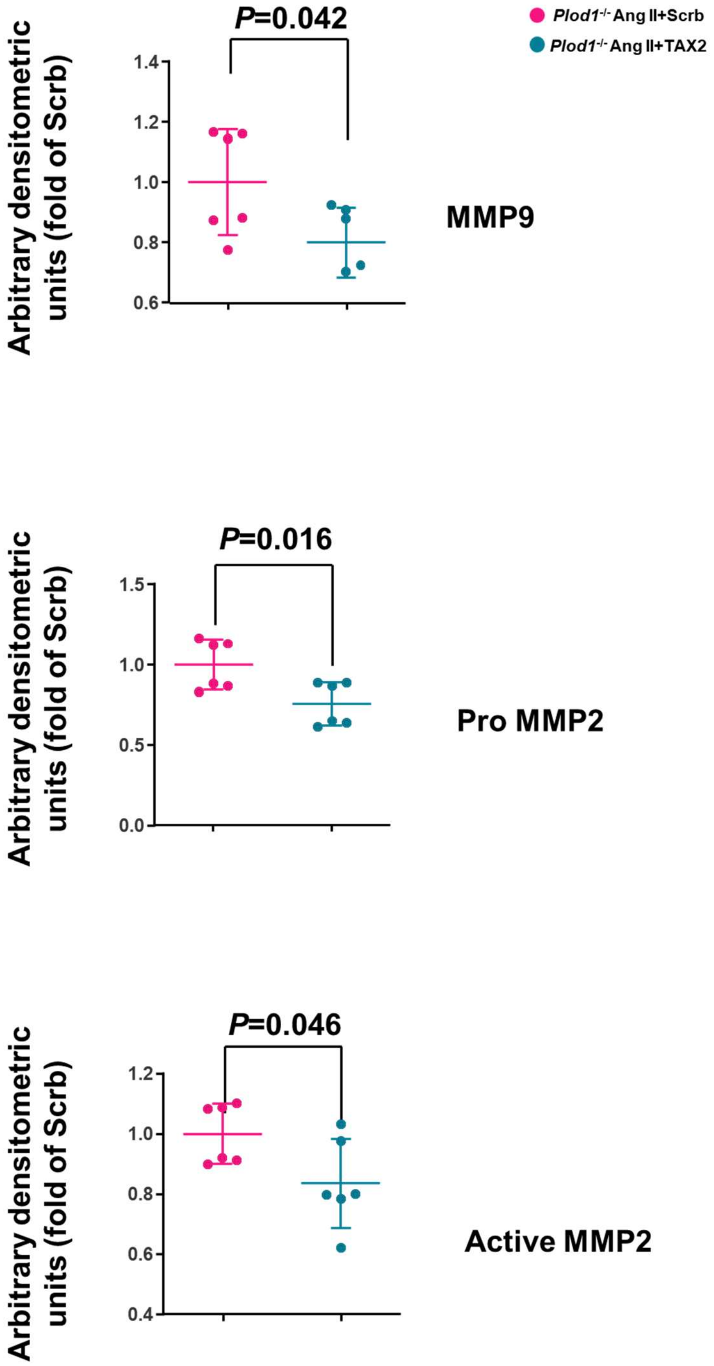

Figure S10. Quantitative analysis of the MMP9, Pro-MMP2 and MMP2 activity in abdominal aortas from *Plod1*$^{-/-}$ mice treated with scrambled peptide (Scrb) or TAX2 followed by angiotensin II (Ang II) infusion for 2 weeks. n = 6. Student's unpaired two-tailed t-test.

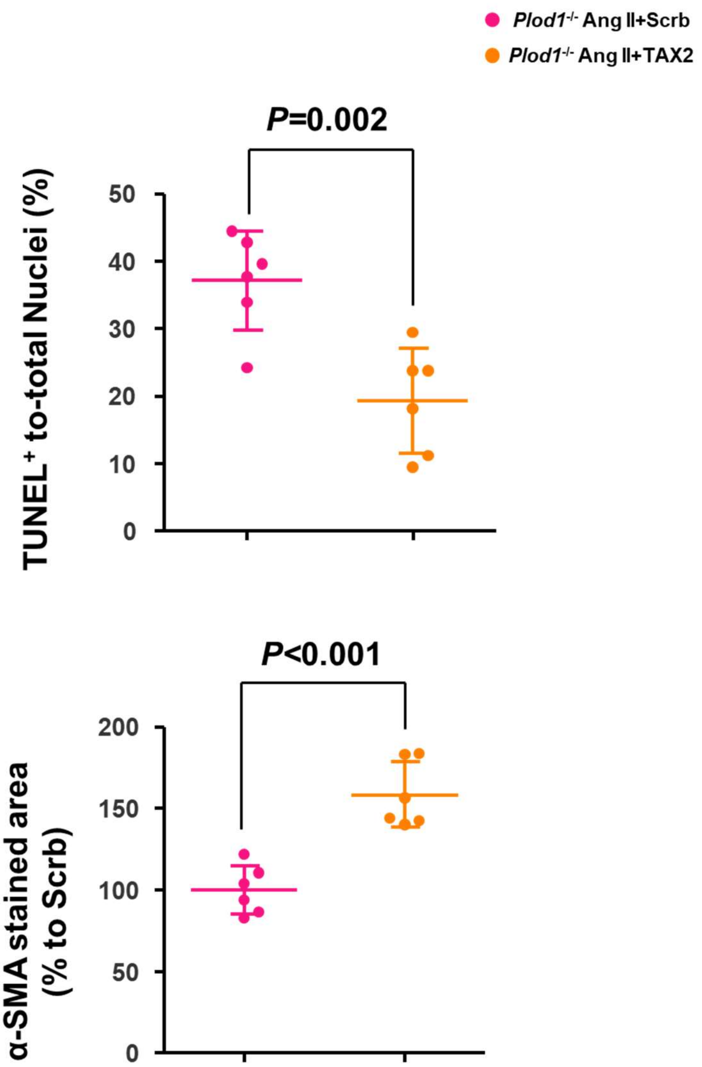

Figure S11. Quantitative analysis of apoptotic cells (upper) and α-SMA (lower) in abdominal aortas from *Plod1*$^{-/-}$ mice treated with scrambled peptide (Scrb) or TAX2 followed by angiotensin II (Ang II) infusion for 2 weeks. n = 6. Student's unpaired two-tailed t-test.

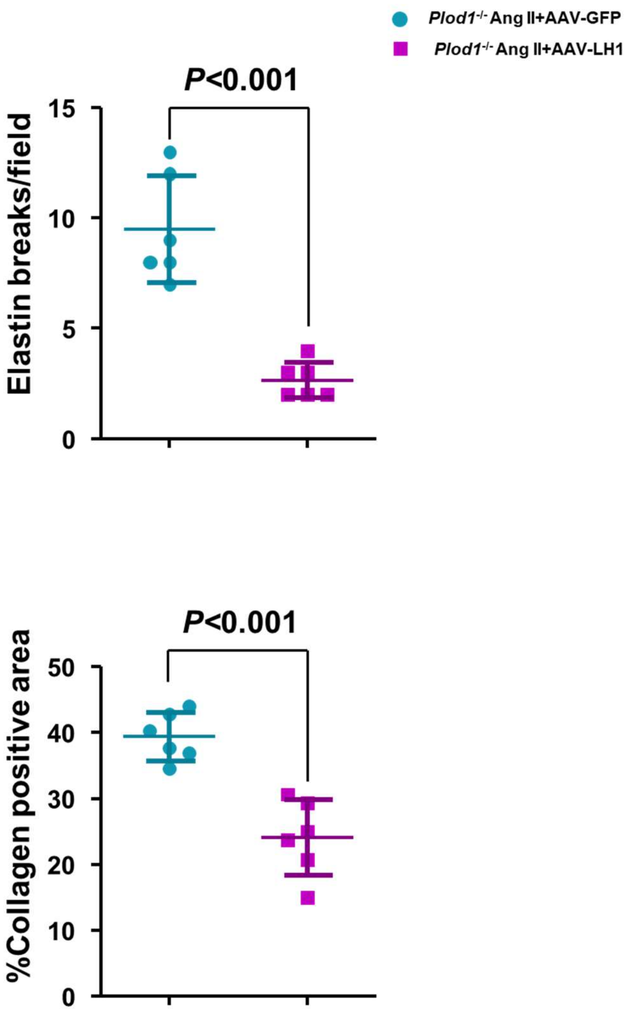

Figure S12. Quantification of elastin breakage (upper) and collagen content (lower) in abdominal aortas from *Plod1*$^{-/-}$ mice treated with AAV-GFP or AAV-LH1 followed by angiotensin II (Ang II) infusion for 2 weeks. n = 6. Student's unpaired two-tailed t-test.

Supplementary Tables

Table S1: Clinical information for the human aorta specimens.

	**Human healthy (non-aneurysmal) aorta (n = 6)**						**Human abdominal aortic aneurysm (n = 6)**					
**SAMPLE#**	#1	#2	#3	#4	#5	#6	#1	#2	#3	#4	#5	#6
**Age (years)**	51	58	45	61	10	57	47	48	64	71	44	66
**Sex**	M	M	M	M	M	M	M	M	M	M	M	M
**Aneurysm Size** (Aortic diameter in mm)	NA	NA	NA	NA	NA	NA	45	58	62	82	67	73
**Comorbidities**												
Smoking	NO	NO	NO	YES	NO	NO	YES	NO	NO	YES	NO	NO
Obesity	NO	NO	NO	NO	NO	NO	NO	NO	NO	YES	NO	NO
Family History	NO	NO	NO	NO	NO	NO	NO	NO	NO	NO	NO	NO
Hypertension	NO	NO	NO	YES	NO	NO	YES	YES	NO	YES	NO	NO
Diabetes	NO	YES	NO	NO	NO	NO	NO	NO	NO	NO	YES	NO
**Medications**												
Statins	NO	NO	NO	NO	NO	NO	NO	NO	NO	NO	NO	NO
ACE Inhibitors	NO	NO	NO	YES	NO	NO	YES	NO	NO	YES	NO	NO
ARBs	NO	NO	NO	NO	NO	NO	NO	NO	NO	NO	NO	NO
Beta Blockers	NO	NO	NO	NO	NO	NO	NO	NO	NO	NO	NO	NO
Antiplatelets	NO	NO	NO	NO	NO	NO	YES	NO	NO	NO	NO	NO

M = male; ACE = Angiotensin converting enzyme; ARB = Angiotensin Receptor Blocker.

Table S2. Sequence of each primer used in real-time PCR

Gene Symbol	Sequence
Il6	Forward: 5’-TAGTCCTTCCTACCCCAATTTCC-3’<br>Reverse: 5’-TTGGTCCTTAGCCACTCCTTC-3’
Ccl2	Forward: 5’- TTAAAAACCTGGATCGGAACCAA-3’<br>Reverse: 5’- GCATTAGCTTCAGATTTACGGGT-3’
Gapdh	Forward: 5’- CTCATGACCACAGTCCATGC -3’<br>Reverse: 5’- CACATTGGGGGTAGGAACAC -3’
